# Immersive Technologies in Sport Spectatorship: A Scoping Review of Psychological Responses and Behavioral Intentions

**DOI:** 10.3390/bs16101903

**Published:** 2026-10-14

**Authors:** Yiming Xiong, Daehwan Kim, Kyungun Kim

**Affiliations:** 1 Department of Marine-Sports, Pukyong National University, Busan 48513, Republic of Korea; xiongym1213@gmail.com; 2 Department of Marketing and Sport Management, University of Central Missouri, Warrensburg, MO 64093, USA; kykim@ucmo.edu

**Keywords:** immersive technology, sport spectatorship, virtual reality, augmented reality, mixed reality, 360-degree video, behavioral intentions, scoping review

## Abstract

Immersive technologies are reshaping sport spectatorship by changing how spectators perceive events, allocate attention, experience emotions, and form behavioral responses. Yet empirical research on virtual reality (VR), augmented reality (AR), mixed reality (MR), and 360-degree video remains fragmented in terms of technologies, theories, methods, and outcomes. This scoping review maps research on immersive technologies in sport spectatorship, focusing on experiential and psychological responses, behavioral intentions, and future research directions. Guided by PRISMA-ScR, we searched Scopus, Web of Science, and ProQuest for English-language peer-reviewed journal articles and supplemented the database search with a citation search. Thirty-four empirical journal articles met the inclusion criteria. VR was the most frequently examined technology. AR has been the subject of a growing amount of research recently. MR and 360-degree video were both underrepresented in the reviewed literature. Most articles used quantitative designs, particularly with post-exposure and online surveys, and were concentrated in a few countries and participant groups. The literature addressed adoption or resistance, immersive experience, psychological responses, and behavioral intentions. The reviewed articles examined experiential constructs such as presence, telepresence, flow, and engagement; cognitive responses such as attention and cognitive load; affective responses such as arousal and enjoyment; evaluative responses such as satisfaction and attitude; and negative responses such as irritation. Overall, the reviewed literature primarily examined the associations between immersive technology types and technology-related features, spectators’ experiential and psychological responses, and behavioral intentions such as continuance intention, word of mouth, revisit intention, intention to pay. Evidence concerning actual long-term spectator behavior remains limited.

## 1. Introduction

Immersive technologies are increasingly reshaping how people encounter, interpret, and respond to mediated environments. Broadly, these technologies refer to digital media systems that blur the boundary between physical and virtual worlds by enriching users’ sensory inputs and enabling more vivid, interactive, and presence-oriented experiences (Suh & Prophet, 2018). In the context of sport spectatorship, immersive technologies such as virtual reality (VR), augmented reality (AR), mixed reality (MR) and 360-degree video have been examined in relation to how spectators watch, understand, and emotionally experience sporting events. VR can place spectators within computer-generated or video-based environments that simulate proximity to the event; AR can overlay real-time digital information onto live or mediated sport settings; MR can integrate virtual objects with physical or mediated environments while allowing interactive engagement with both; and 360-degree video can provide panoramic viewing perspectives that expand spectators’ control over visual attention and viewpoint selection.

These technological affordances are especially relevant to behavioral science because sport spectatorship is not merely a media consumption activity but also a psychologically rich experience involving attention, emotion, identification, satisfaction, social connection, and behavioral intention. Prior studies suggest that immersive sport media can enhance perceived vividness, enjoyment, flow, presence, telepresence, and satisfaction, thereby influencing spectators’ attitudes and intentions toward sport content, teams, events, or technologies (Goebert & Greenhalgh, 2020; Jang et al., 2022; D. Kim & Ko, 2019). For example, VR spectatorship (VRS) has been associated with stronger flow and satisfaction, while AR-enhanced sport broadcasts and event experiences have been examined in relation to perceived usefulness, visual appeal, experience value, word of mouth, revisit intention, and continuance intention. These findings indicate that immersive technologies may create new psychological pathways through which sport organizations and media providers can shape spectator experience and behavior.

However, immersive technologies do not automatically generate positive outcomes. They often intensify sensory stimulation and require spectators to process multiple streams of visual, spatial, and interactive information, which is why they may also produce negative responses such as distraction, irritation, cognitive load, visual fatigue, or discomfort (Ahn & Ko, 2024; Oh et al., 2022). This dual potential is particularly important in sport settings, where spectators must continuously distribute their attention among game action, contextual information, social cues, advertising messages, and technology-enhanced overlays. Therefore, immersive sport spectatorship should be understood not only as a technological innovation but also as a complex behavioral phenomenon in which technological features, individual differences, cognitive processing, emotional responses, and behavioral consequences interact.

Despite the growing number of empirical studies on immersive technologies in sport spectatorship, the literature remains disjointed in terms of technology types, theoretical perspectives, methodological designs, and outcome variables. Although existing studies have examined VR, AR, MR and 360-degree video in relation to technology acceptance, fan experience, psychological response mechanisms, and behavioral intentions, integrative knowledge across these research streams remains limited. Previous reviews of immersive technologies have either examined consumer behavior across broader commercial contexts, in which retail has been particularly prominent, or focused on specific sport applications, technologies, or settings, such as interactive VR in the broader sport domain, digital technologies for football fan interaction, or AR in sporting events. In contrast, the present review focuses specifically on sport spectatorship and integrates evidence across VR, AR, MR, and 360-degree video from a behavioral perspective. Specifically, it remains unclear how immersive technologies have been conceptualized, which theories and methods have been employed, which participant groups and sport contexts have been examined, and which psychological and behavioral science variables have received the greatest attention. This fragmentation makes it difficult to assess the current state of knowledge, identify underdeveloped areas, and develop a coherent behavioral framework for future research.

Accordingly, this study conducts a scoping review of empirical research on immersive technologies in sport spectatorship, spanning 34 empirical journal articles published up to 2026. The review aims to map the development of the field, synthesize the theoretical and methodological characteristics of existing articles, and identify key psychological responses, and behavioral intentions, and related outcomes associated with immersive sport viewing. Additionally, this review aims to clarify the conceptual structure of the field and provide an evidence-based agenda for future behavioral research on immersive sport spectatorship. This study addresses the following research questions:

Q1: How have immersive technologies been conceptualized and operationalized in sport spectatorship research?

Q2: What are the publication trends and major journal outlets in research on immersive technologies and sport spectatorship?

Q3: Which geographical contexts and participant groups have been examined in this field?

Q4: Which theories and research methods have been used to investigate immersive technologies in sport spectatorship?

Q5: What antecedent, experiential and psychological response, moderating, control, and behavioral-intention variables have been examined in relation to immersive sport spectatorship?

Q6: What research gaps and future directions for behavioral research on immersive technologies in sport spectatorship have emerged?

By answering these questions, this review contributes to the literature in three ways. First, it consolidates empirical findings on VR, AR, MR, and 360-degree video in sport spectatorship into a single behavioral science-oriented perspective. Second, it identifies the dominant theoretical lenses, methodological approaches, and variable structures through which immersive sport experiences have been studied. Third, it proposes future research directions for examining the positive and negative psychological mechanisms through which immersive technologies are associated with spectator behavioral intentions. In doing so, this study provides a foundation for researchers seeking to advance theory on immersive media, sport consumer behavior, and technology-mediated spectatorship. It also offers practical insights for sport organizations, broadcasters, event managers, and technology developers seeking to design effective and psychologically sustainable immersive viewing experiences.

## 2. Overview of Immersive Technologies

This section clarifies the conceptual scope of immersive technologies as used in this scoping review. The term “immersive technology” is applied inconsistently across VR, AR, MR 360-degree video, mixed reality, and other interactive media systems. Therefore, specifying which technologies are included and how they are distinguished in the context of sport spectatorship is necessary. This review defines immersive technologies as media technologies that enrich spectators’ sensory, spatial, and interactive experiences of sport events and thereby have the potential to influence psychological mechanisms and behavioral outcomes.

### 2.1. Defining Immersive Technologies

**Figure 1:**
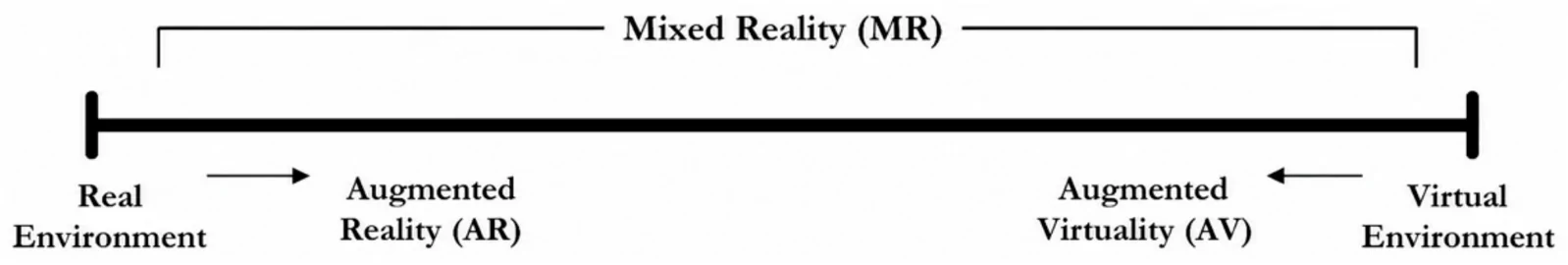
The reality–virtuality continuum and focal immersive technologies in sport spectatorship (Milgram et al., 1995).

Scholars have defined immersive technologies from several perspectives. Slater (2009) emphasized the quality and quantity of sensory information provided to users, treating immersion as a system-level property that can support the feeling of being in a mediated environment. Suh and Prophet (2018) similarly described immersive technologies as digital systems that blur the boundary between physical and virtual environments by enhancing users’ sensory experience. The reality–virtuality continuum proposed by Milgram et al. (1995) is useful for locating different immersive technologies along a spectrum ranging from the physical environment to fully virtual environments (Figure 1). Within this continuum, AR, VR, and related technologies do not represent a simple hierarchy of more or less immersion; rather, they create immersion through different combinations of sensory richness, spatial representation, and interactivity.

It is also important to distinguish technological immersion from psychological responses to immersion (D. Kim & Ko, 2019). Immersion refers to the objective affordances of a media system, such as sensory fidelity, field of view, interactivity, and environmental substitution. By contrast, presence, telepresence, flow, engagement, enjoyment, and cognitive absorption refer to spectators’ subjective responses to that system. This distinction is central to behavioral research because highly immersive media do not necessarily produce positive psychological or behavioral outcomes.

Based on these definitions, this review defines immersive technologies in sport spectatorship as media technologies that enhance the spectator’s viewing experience by augmenting sensory input, spatial perspective, interactivity, or perceived proximity to a sport event. The operational scope of this review includes VR, AR, MR and 360-degree video because these three technologies have been directly examined in empirical research on sport spectatorship and spectator behavior (Du et al., 2024; Jang et al., 2022; D. Kim & Ko, 2019). Technologies used exclusively for athlete training, coaching, rehabilitation, sport performance analysis, officiating, or general engineering development are outside the scope of this review unless they explicitly examine spectators’ psychological or behavioral responses to sport viewing.

### 2.2. Virtual Reality and Sport Spectatorship

VR is a technology that creates a computer-generated or video-based simulated environment in which users can perceive and interact with a mediated world (Suh & Prophet, 2018). VR differs from other immersive media because it can extensively replace users’ perception of the physical environment with a digitally mediated environment. The central aim of VR is to make users feel as though they are located within the environment generated or represented by the system (Slater & Sanchez-Vives, 2016).

VR can be classified into non-immersive and immersive forms. Non-immersive VR is usually experienced through a conventional screen and traditional input devices, whereas immersive VR commonly involves head-mounted displays (HMDs) that provide a wider field of view, stronger sensory enclosure, and greater perceived spatial proximity. In the broader sport context, Neumann et al. (2018) described interactive VR as a simulated environment that allows users to engage with sport-related activities and experience a sense of presence.

In sport spectatorship, D. Kim and Ko (2019) defined VRS as watching sport events in a mediated environment through VR technology. VR sport spectatorship can increase the depth and breadth of sensory information and provide interactive user interfaces that allow spectators to experience sport content from perspectives that are difficult to access through conventional broadcasting. Existing studies have used VR sport spectatorship to examine technology acceptance, presence, telepresence, flow, satisfaction, cognitive and emotional processing, brand memory, and behavioral intention (D. Kim & Ko, 2019; Lee & Zhou, 2025; Oh et al., 2022; Paek et al., 2025; Walton, 2025).

However, VRS is not necessarily unidirectionally positive. It may also produce negative effects, such as cybersickness and visual fatigue, particularly when users are exposed to VR environments for an extended period (Caserman et al., 2021). Oh et al. (2022) further noted that the enjoyment derived from VRS may decline over time because users can adapt to repeated or prolonged exposure to positive stimuli.

This review therefore treats VR as a form of immersive sport spectatorship in which the spectator’s viewing environment is substantially reconstructed through a virtual or video-based spatial environment. Its relevance to behavioral research lies in the way VR has been examined in relation to spectators’ sense of presence, attention allocation, emotional arousal, satisfaction, and subsequent behavioral intentions. The literature also points to the need to examine negative responses and the factors that may reduce their consequences.

### 2.3. Augmented Reality and Sport Spectatorship

AR is technology that overlays virtual objects, graphics, or information onto a real or mediated environment. Unlike VR, AR does not replace the physical environment; rather, it enhances it by adding contextually relevant digital information (Azuma, 1997). Classic definitions of AR emphasize three features: the combination of real and virtual elements, real-time interactivity, and spatial registration between digital content and the user’s environment (Azuma, 1997). In this sense, AR occupies a different position from VR on the reality–virtuality continuum because it maintains the spectator’s connection to the real sport environment while adding digital layers of information or experience.

In sport spectatorship, AR can be implemented in multiple settings, including sport broadcasting, in-stadium viewing, mobile applications, HMD-based systems, and projector-based displays (Schmalstieg & Hollerer, 2016). Prior research has examined the use of AR in sport marketing, sport broadcasting, live streaming, mega-sport events, and stadium-based fan experiences (Ahn & Ko, 2024; Du et al., 2024; Goebert et al., 2022; Goebert & Greenhalgh, 2020; Lei & Ngan, 2024; Ngan & Lei, 2025; Rogers et al., 2017; Uhlendorf & Uhrich, 2024). Across these contexts, AR has been investigated as a technology that can enhance visual appeal, perceived usefulness, information quality, experience value, enjoyment, perceived autonomy, competence, relatedness, revisit intention, word of mouth, and continuance intention.

However, AR may also create perceptual and cognitive challenges. It adds information to an already complex sport-viewing environment. Such information may support spectators’ understanding and enjoyment, but it may also generate irritation, distraction, sensory overload, and resistance when it is poorly timed, visually intrusive, or misaligned with spectators’ goals. (Ahn & Ko, 2024; Rogers et al., 2017; Uhlendorf & Uhrich, 2024). This dual role makes AR especially relevant to behavioral science: AR sport spectatorship is not only a question of whether digital overlays are technically feasible, but also of how spectators attend to, interpret, accept, resist, and act upon augmented information.

### 2.4. Mixed Reality and Sport Spectatorship

Mixed reality (MR) combines physical or mediated environments with digitally generated objects, allowing users to interact with both real and virtual elements. Unlike VR, MR does not fully replace the surrounding environment, while its digital content may be more spatially integrated and interactive than conventional AR. However, MR remains underrepresented in sport spectatorship research. Only one included article examined sport fans’ adoption of a mixed-reality headset in relation to technology acceptance, team identification, and purchase intention (Selvaratnam et al., 2026).

### 2.5. 360-Degree Video and Sport Spectatorship

360-degree video is produced using omnidirectional cameras or multi-camera systems that capture a spherical field of view. Viewers can navigate the visual scene by rotating their viewpoint through a computer, smartphone, tablet, or HMD (Snelson & Hsu, 2020). Although 360-degree video is often discussed together with VR because both can generate immersive viewing experiences, the two technologies should be analytically distinguished. VR usually refers to a digitally generated or substantially simulated environment where users may interact with virtual elements, whereas 360-degree video typically records real-world scenes and allows users to control the viewing direction without necessarily manipulating the environment itself (Kittel et al., 2020; Roche et al., 2025; Snelson & Hsu, 2020).

From a technological perspective, 360-degree video differs from fully interactive VR in that users primarily control the direction of view rather than the structure or behavior of the environment. These characteristics may have implications for spatial perspective, viewing autonomy, presence, and engagement; however, the empirical evidence in sport spectatorship is currently too limited to draw conclusions regarding these effects.

Sport spectatorship literature has focused less on 360-degree video than on VR and AR. Only one 360-degree video article was identified in the present review. As one of the few studies on 360-degree video in sport spectatorship, Jang et al. (2022) examined how high-tech camera technology influenced sport consumers’ viewing experiences and behavioral intentions. In this article, 360-degree video was examined in relation to perceived novelty, distraction, presence, enjoyment, credibility, and behavioral intentions. However, these findings should be interpreted as preliminary and indicative rather than as evidence of general or stable effects of 360-degree video. Given the few studies in this area, the present review treats 360-degree video as a distinct but underdeveloped form of immersive sport spectatorship.

### 2.6. Conceptual Boundary for the Scoping Review

VR, AR, MR and 360-degree video represent different technological pathways to immersive sport spectatorship. VR primarily reconstructs the viewing environment; AR augments an existing live or mediated sport environment; and 360-degree video expands spectators’ spatial perspective and viewpoint control. Despite these differences, all three technologies are relevant to this scoping review because they can alter how spectators perceive, attend to, emotionally experience, evaluate, and behaviorally respond to sport events.

Therefore, this review uses “immersive technologies” as an umbrella term for VR, AR, MR and 360-degree video applications that are explicitly connected to sport spectatorship. This scope is intentionally narrower than the broader field of immersive sport technology, which also includes training simulations, athlete performance systems, rehabilitation tools, officiating systems, and technical prototypes. By establishing this conceptual boundary, the review focuses on studies that examine spectators as users, consumers, fans, or audiences, and on ones that report psychological, experiential, or behavioral outcomes associated with immersive sport viewing.

## 3. Methods

### 3.1. Review Design and Reporting Framework

This study employed a scoping review design to map the scope, characteristics, theoretical foundations, methodological approaches, and variable structures of empirical research on immersive technologies in sport spectatorship. A scoping review is appropriate because research on immersive technologies in sport spectatorship is ongoing, conceptually heterogeneous, and dispersed across technology types, theoretical perspectives, methods, and outcome variables. Accordingly, this review aimed to clarify the structure of the field, identify dominant research patterns, and develop an evidence-based agenda for future behavioral research.

The review was guided by Arksey and O’Malley’s (2005) scoping review framework, refined by Levac et al. (2010), and reported in accordance with the PRISMA Extension for Scoping Reviews (PRISMA-ScR; Tricco et al., 2018). The review process consisted of six stages: identifying the research questions, identifying relevant articles, selecting articles, charting the data, collating and synthesizing the results, and reporting implications for future research. The review was not prospectively registered. Nevertheless, the research questions, eligibility criteria, information sources, search strings, screening procedure, and charting categories were specified a priori and applied consistently throughout article selection, data charting, and synthesis.

The eligibility criteria were structured using the population–concept–context framework. The population comprised sport spectators, fans, audiences, viewers, and consumers. The concept was immersive technology, operationalized as virtual reality (VR), augmented reality (AR), 360-degree video, extended reality (XR), and mixed reality (MR). Related terms, including immersive media, immersive video, and spatial computing, were additionally used to maximize the retrieval of relevant studies. The context was sport spectatorship, including mediated, remote, live, recorded, and on-site sport-viewing experiences.

### 3.2. Search Strategy and Information Sources

A systematic search was conducted in three major academic databases: Scopus, Web of Science, and ProQuest. These databases were selected because they provide broad coverage of peer-reviewed research in sport management, communication, marketing, psychology, consumer behavior, and technology-related fields. The search was completed on 10 July 2026, and no lower date limit was applied.

Before the final database search, preliminary searches were conducted to identify relevant keywords and refine the search strategy. The final search strategy combined four term blocks: technology-related terms, sport-related terms, spectator/viewing-related terms, and experience/behavior-related terms. Specifically, the technology terms were “augmented reality” OR AR OR “virtual reality” OR VR OR “360-degree” OR “extended reality” OR XR OR “mixed reality” OR MR OR “immersive media” OR “immersive video” OR “spatial computing”; the sport term was “sport*”; the spectator/viewing-related terms were spectator* OR view* OR watch* OR fan* OR audience*; and the experience/behavior-related terms were experience* OR spectatorship* OR behavior* OR intention* OR engagement* OR behaviour* (Table 1).

The search was adapted to the syntax and searchable fields of each database. Searches were conducted in title, abstract, keyword, topic, or abstract fields depending on database functionality. Only English-language peer-reviewed journal articles were retained. Conference papers, books, book chapters, dissertations, reports, non-peer-reviewed publications, and non-English publications were excluded at this stage.

The initial search yielded 842 records before database filters were applied. After limiting the results to English-language journal articles, 355 records remained: 142 from Scopus, 116 from Web of Science, and 97 from ProQuest.

### 3.3. Eligibility Criteria

Articles were included if they met all the following criteria: they were peer-reviewed empirical journal articles; were written in English; examined VR, AR, or 360-degree video, XR, or MR; were situated in a sport spectatorship, sport viewing, fan experience, or sport audience context; and reported spectator-related psychological, experiential, attitudinal, or behavioral outcomes. Eligible articles included quantitative, qualitative, and mixed-methods empirical designs, although only one exclusively qualitative article was identified in the final sample.

Articles were excluded if they met any of the following criteria: they discussed immersive technologies only in a general or conceptual manner; were unrelated to sport spectatorship; focused primarily on athlete training, coaching, sport education, physical activity, fitness, rehabilitation, officiating, or sport performance analysis; were purely technical or engineering studies without spectator-related psychological or behavioral outcomes; used immersive technology only as an experimental manipulation without examining spectator experience or behavior; examined immersive technology primarily as a clinical or therapeutic exposure medium rather than as a sport-viewing technology (e.g., VR exposure therapy for social anxiety; Chung et al., 2024); were reviews, meta-analyses, literature surveys, conference papers, books, dissertations, reports, or other non-empirical publications; or were not English-language peer-reviewed journal articles.

Borderline cases were evaluated according to the conceptual boundary established in Section 2; the article had to examine spectators, fans, viewers, audiences, sport consumers, or event attendees as the focal users of immersive sport-viewing technology.

### 3.4. Articles Selection and Inclusion Process

Articles selection was conducted by two researchers using the predefined eligibility criteria. The two researchers independently screened the titles and abstracts of the retrieved records and subsequently assessed the full texts of potentially eligible articles. Any disagreements regarding eligibility were resolved through discussion until consensus was reached. A total of 138 duplicates were removed from the 355 database records, leaving 217 unique records for title and abstract screening. Records were excluded if they clearly did not meet the eligibility criteria. The most common reasons for exclusion were lack of relevance to immersive technology, lack of relevance to sport spectatorship, focus on athlete training or sport performance rather than spectators, technical or engineering focus without spectator outcomes, non-empirical design, or lack of relevance to spectator experience or behavior. After title and abstract screening, 34 articles were retained for full-text assessment.

The full texts of the 34 articles were assessed against the inclusion and exclusion criteria. Seven articles were excluded at this stage for three main reasons. Two articles did not examine sport spectatorship supported by VR, AR, or 360-degree, XR, or MR technologies: Huang et al. (2026) focused on virtual character consumption, and Zhang et al. (2025) examined smart stadiums. Three articles applied immersive technology to athletes or exercisers rather than to spectators: Uhm et al. (2026) employed VR primarily for athletes’ participation and competitive performance, with spectators merely watching athletes compete in a virtual environment; Luttmann and Jansen (2026) examined the effects of VR on cycling exercise; and Hollaus et al. (2025) developed and evaluated a VR simulator for luge training with athletes and domain experts. Two articles used immersive technology only instrumentally rather than as the focus of inquiry: Chang and Inoue (2021) used VR solely as a tool for presenting experimental stimuli, with the central research question concerning how game outcomes and competitive contexts influenced spectators’ emotions and psychological vitality, and Zhu (2025) regarded VR as a sport promotion and advertising medium that was examined for its effects on sport interest and participation intention rather than on immersive sport-viewing experiences. Following full-text assessment, 27 articles were retained from the database search.

To reduce the risk of omitting relevant studies, supplementary identification was conducted after the database-centered selection process. First, backward citation chasing was performed by reviewing the reference lists of the included articles. Second, targeted Google Scholar searches were conducted using the same core keyword combinations. Retrieved records were assessed using the same eligibility criteria as the database records. Seven additional eligible articles were identified, resulting in a final sample of 34 empirical articles.

**Figure 2:**
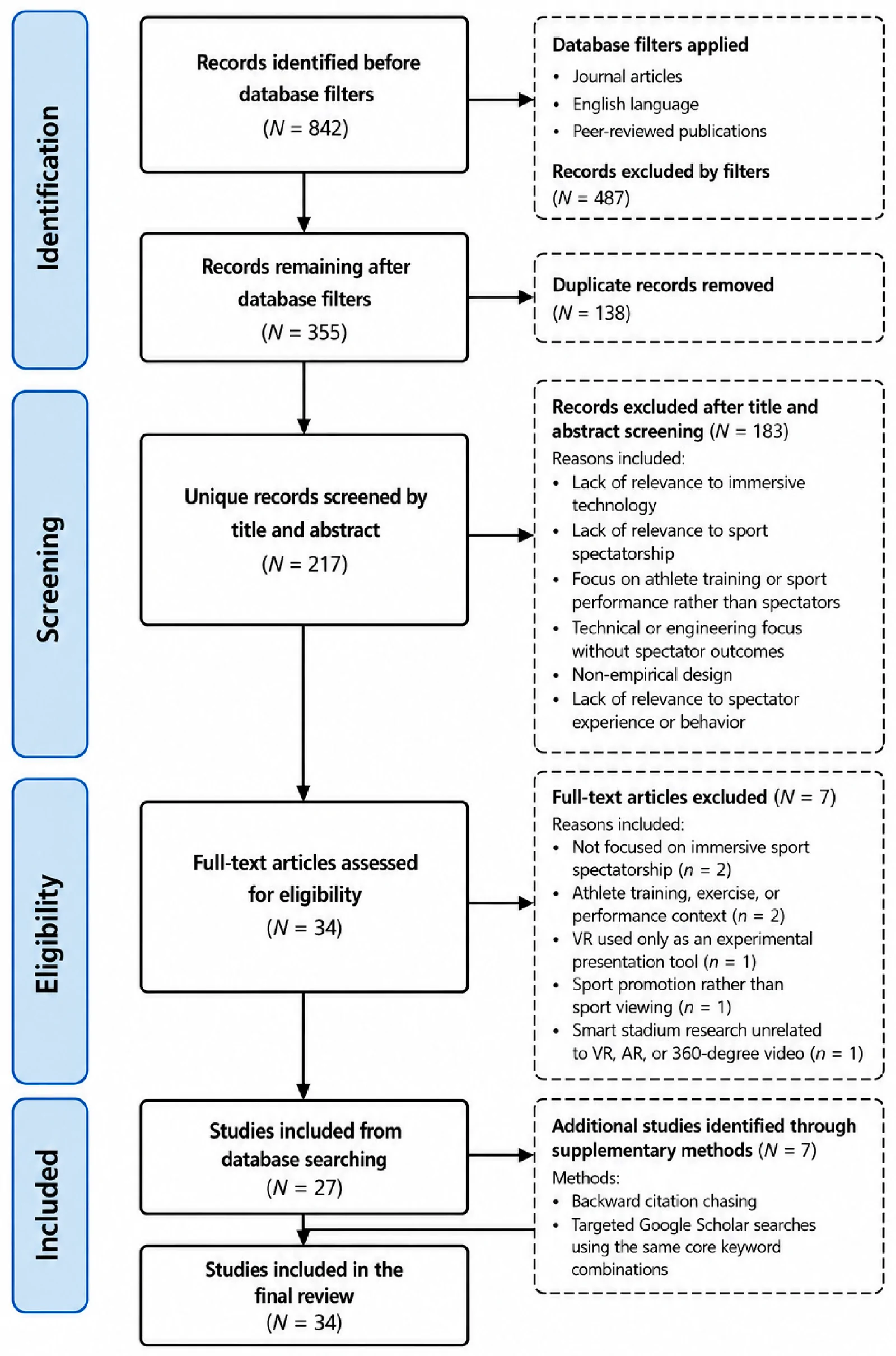
PRISMA-ScR flow diagram of the study selection process.

Figure 2 presents the PRISMA-ScR flow diagram for the study selection process. A total of 842 records were identified before database filters, 355 records remained after filters, 138 duplicates were removed, 217 titles and abstracts were screened, 34 full texts were assessed, 7 full-text articles were excluded, 27 articles were retained from database searching, 7 additional articles were identified through supplementary search and citation chasing, and 34 articles were included in the final review.

### 3.5. Data Charting and Synthesis

A data charting form was developed in Microsoft Excel to extract and organize information from the articles. The charting form included 19 categories: author(s), publication year, journal, technology type, device or interface, sport and viewing context, country, participant characteristics, sample size, research methods, data collection methods, research theme, theories or models, independent variables, mediators, dependent variables, moderators, control variables and key findings. To ensure consistency in data charting and classification, two researchers were involved in the coding process. Before formal coding, the researchers pilot-coded a subset of the included articles and discussed the coding items, category boundaries, and decision rules. Based on these discussions, the coding framework was refined before being applied to the full set of articles. The classification of research themes, theoretical frameworks, research methods, variable roles, and key findings was then independently coded by the two researchers. Any discrepancies were resolved through discussion until consensus was reached. The descriptive and methodological characteristics of the included articles, including the publication outlet, technology type, device or interface, sport and viewing context, country, participant characteristics, sample size, research methods, and data collection methods, are presented in Appendix A (Table A1). Participant and sample characteristics, including participant characteristics, sample size, and the reporting of age, gender, and prior VR/AR/MR/360-degree experience, are summarized in Appendix A (Table A2). Research themes, theoretical frameworks or models, variable structures, and key findings are presented in Appendix A (Table A3).

To avoid conflating “articles” with “independent studies,” this review treated the included peer-reviewed journal articles as the primary reporting units and distinguished the number of articles from the number of independent studies. An article was defined as a peer-reviewed journal article published as an independent publication with clearly identified authors, title, journal, and publication year. An independent study was defined as an investigation within an article that had a relatively distinct research objective, research design, and participant sample. The determination of study independence was based on whether the original article clearly distinguished different research objectives, research designs, or participant samples, together with whether the studies used independent participant samples. Labels used in the original articles, such as “Study 1,” “Study 2,” “Experiment 1,” or “Experiment 2,” were used as supporting evidence when determining study independence. When an article contained multiple studies, each study was separately identified and coded within the corresponding article entry in the appendices. When the same variable appeared in different independent studies, it was recorded according to its specific role in each study’s analytical model but was not counted as a different variable.

The extracted data were synthesized using descriptive mapping and narrative thematic synthesis. First, descriptive mapping was used to summarize publication trends, journal outlets, geographical distribution, participant characteristics, research methods, and technology types. Second, theoretical mapping was used to identify the theories and models applied across the included articles. Third, thematic synthesis was used to group the articles according to their primary focus, including adoption of or resistance to immersive technology, immersive technology experiences, psychological response mechanisms, and related outcomes. Finally, variable mapping was used to classify antecedent, experiential and psychological response, moderating, control, and outcome variables, including behavioral intentions, examined in the included articles. These elements were subsequently combined in a proposed integrative evidence map summarizing the relationships examined across the literature, including technology-related features, experiential and psychological responses, negative responses, conditional factors, and behavioral intentions or related outcomes.

## 4. Results

This section presents the results of the scoping review in three parts. First, descriptive mapping summarizes publication trends, journal outlets, geographical distribution, participant characteristics, and research methods. Second, theoretical mapping identifies the major theories and models applied across the included articles to examine immersive technologies in sport spectatorship. Third, thematic and variable mapping synthesizes the primary research themes, antecedents, experiential and psychological responses, negative responses, behavioral intentions, and conditional factors, and control variables examined in the included articles. These elements are subsequently organized into a proposed evidence map that visually represents the overall structure of the existing evidence on immersive sport spectatorship and highlights the principal relationships examined in prior research.

### 4.1. Descriptive Mapping of the Articles

#### 4.1.1. Publication Trends

**Figure 3:**
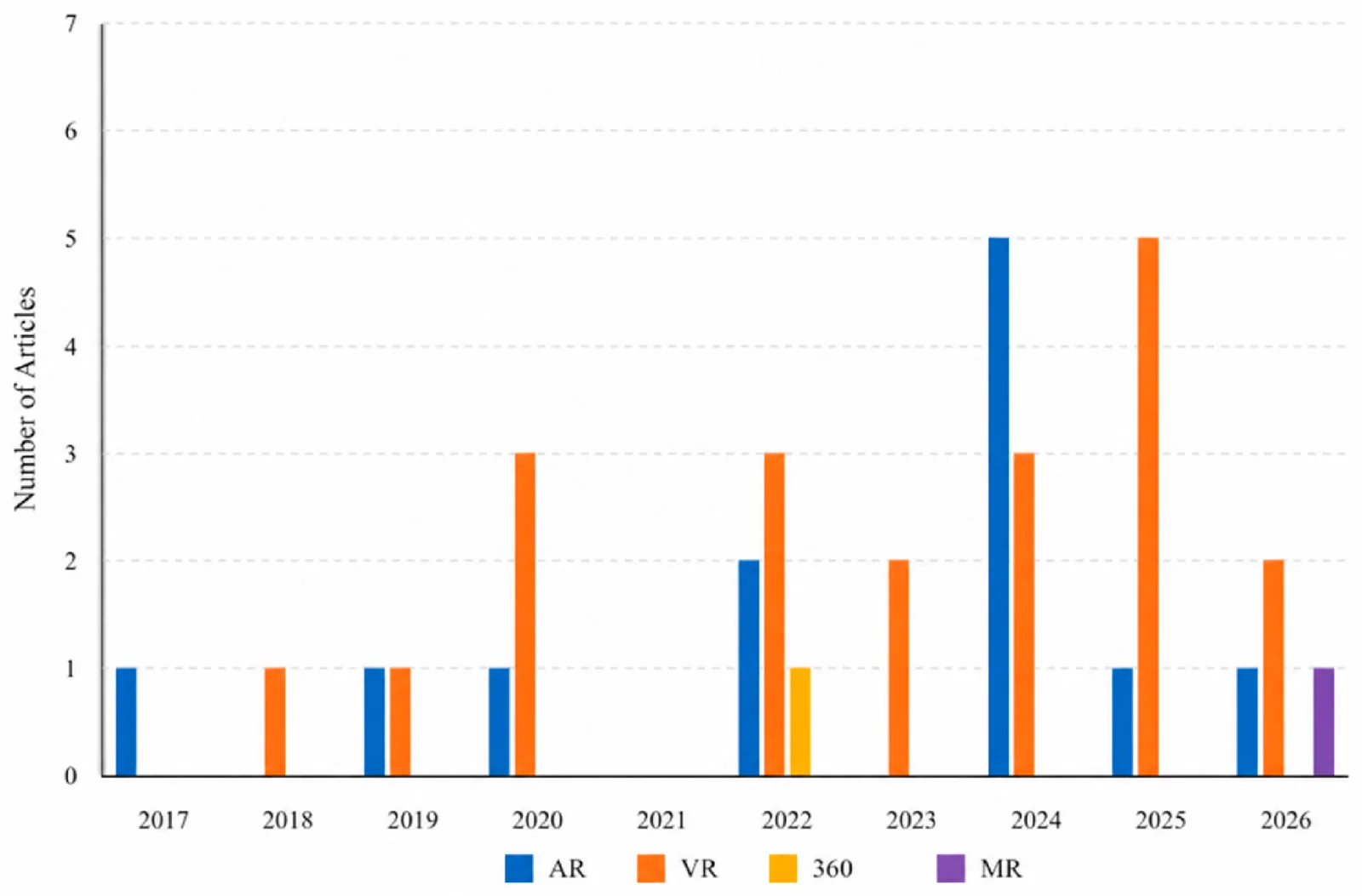
Publication trends of the included articles by year and technology type.

Figure 3 presents the annual distribution of the 34 articles according to each article’s final bibliographic publication year. Overall, empirical research on immersive technologies in sport spectatorship has increased over time, although the pattern is not strictly linear. The annual output peaked in 2024, with eight articles, followed by six articles in 2025. AR publications peaked in 2024 with five articles, whereas VR publications reached their highest annual output in 2025 with five articles. None of the articles were published in 2021, whereas four articles were identified for 2026. Because the 2026 literature represents only a partial publication year, its frequency should not be interpreted as indicating a decline in research activity.

At the study level, the 34 included articles comprised 40 independent studies. Of these, 22 examined VR, 16 examined AR, one examined MR, and one examined 360-degree video. VR therefore represented the largest body of empirical research, while AR also accounted for a substantial proportion of the included studies. By contrast, MR and 360-degree video were each examined in only one independent study. This distribution indicates that the literature has expanded primarily around VR and AR, while empirical evidence on MR and 360-degree video remains limited.

#### 4.1.2. Publication Outlets

Table 2 summarizes the journal outlets in which the 34 included articles were published. These articles appeared across 24 journals, indicating that research on immersive technologies in sport spectatorship is dispersed across sport management, sport marketing, communication, consumer behavior, human–computer interaction, tourism, and technology-related outlets.

The most frequent outlet was the *International Journal of Sports Marketing and Sponsorship* (*n* = 5), followed by *Computers in Human Behavior* (*n* = 4), *Frontiers in Virtual Reality* (*n* = 2), and *Sport Marketing Quarterly* (*n* = 3). The remaining journals each published one included article. This distribution suggests that immersive sport spectatorship has been examined not only as a sport management or sport marketing topic, but also as a broader interdisciplinary issue involving technology-mediated experience, consumer response, and digital behavior.

#### 4.1.3. Geographical Distribution

**Figure 4:**
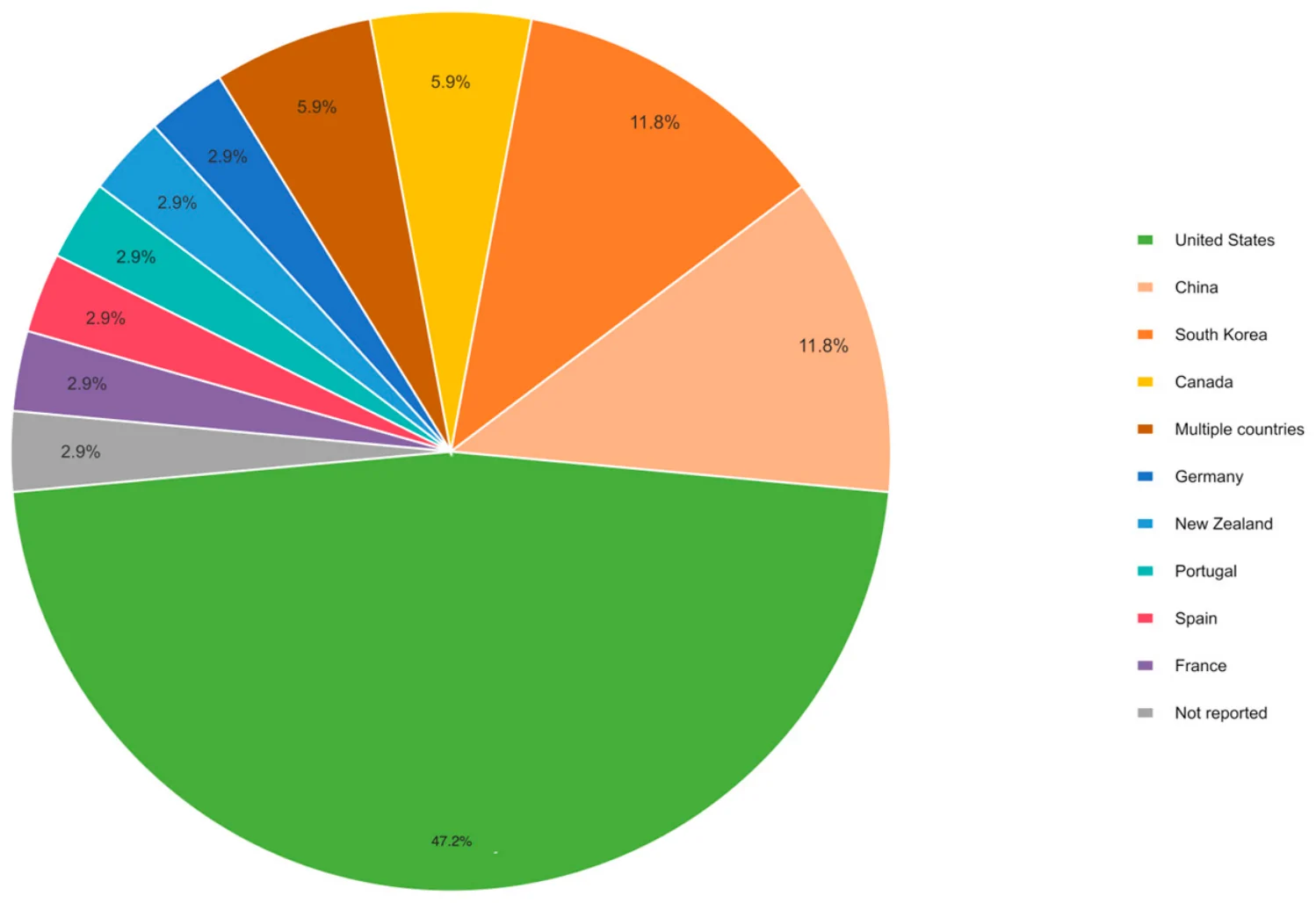
Geographical distribution of the included articles.

Figure 4 shows the geographical distribution of the included articles. The United States was the most frequently represented research context, accounting for 16 articles, or approximately 47.2% of the included articles. China accounted for four articles, South Korea for four articles, and Canada for two articles. Two articles involved multiple countries. Germany, New Zealand, Portugal, France, and Spain were each represented by one article, while the location was not reported for one article.

The distribution indicates that research is strongly concentrated in a small number of national contexts, particularly the United States (*n* = 16). Asian contexts such as China (*n* = 4) and South Korea (*n* = 4) are increasingly represented, whereas evidence from other regions remains limited. This concentration should be considered when interpreting the generalizability of existing findings on immersive sport spectatorship.

#### 4.1.4. Participant Characteristics

Table 3 summarizes the participant characteristics reported in the 34 included articles. University samples were the most common (*n* = 17), followed by sport-related samples (*n* = 8), general population samples (*n* = 3), context-specific samples (*n* = 3), and mixed samples (*n* = 3).

Seventeen of the 34 articles (50.0%) relied exclusively on university student samples. An additional two articles (5.9%) included university students as part of mixed samples. Thus, university students were represented in 19 of the 34 articles (55.9%). However, this percentage reflects representation at the article level rather than the proportion of students among all participants, because the mixed-sample articles did not report student-specific sample sizes.

Appendix A (Table A2) provides article-level participant and sample information for all 34 included articles, including participant characteristics, sample size, and whether age, gender, and prior VR/AR/MR/360-degree experience were reported. Age information was reported in 31 of the 34 articles (91.2%), and gender information was reported in 33 articles (97.1%). Prior immersive-technology experience was reported for the full sample in 17 articles (50.0%), while one additional article reported this information for only one of its two studies.

The frequent use of university student samples indicates that the existing evidence remains substantially concentrated in student populations. At the same time, several articles recruited sport fans, spectators, event attendees, general-population participants, and participants with prior immersive-technology or sport-viewing experience, indicating some diversity in participant recruitment. However, the evidence base remains limited with respect to older adults, casual spectators, families, international sport tourists, disabled spectators, and long-term users of immersive sport-viewing technologies.

#### 4.1.5. Sport and Viewing Contexts

Detailed information on the sport and viewing context of each article is presented in Appendix A (Table A1). The articles examined immersive sport-viewing technologies in different sporting contexts, including basketball, football (soccer), American football, tennis, volleyball, rugby, baseball, luge, and Olympic or multisport events. Laboratory-based viewing was identified in 11 of the 34 articles (32.4%). Ten articles used laboratory viewing exclusively, whereas one article combined laboratory-based simulated viewing with on-site stadium viewing. These laboratory-based articles typically presented prerecorded game clips or highlights under controlled viewing conditions. Beyond laboratory settings, the reviewed articles also examined online viewing, on-site or field-based viewing, live streaming, and prior actual use of immersive sport-viewing technologies. Several articles did not involve direct use of the technology but instead presented hypothetical or conceptual sport-viewing scenarios through descriptions, images, or demonstration videos. Overall, the evidence base was dominated by short-duration and controlled or mediated viewing contexts, with comparatively limited research examining sustained real-world use and live spectator experiences.

#### 4.1.6. Research Methods

**Figure 5:**
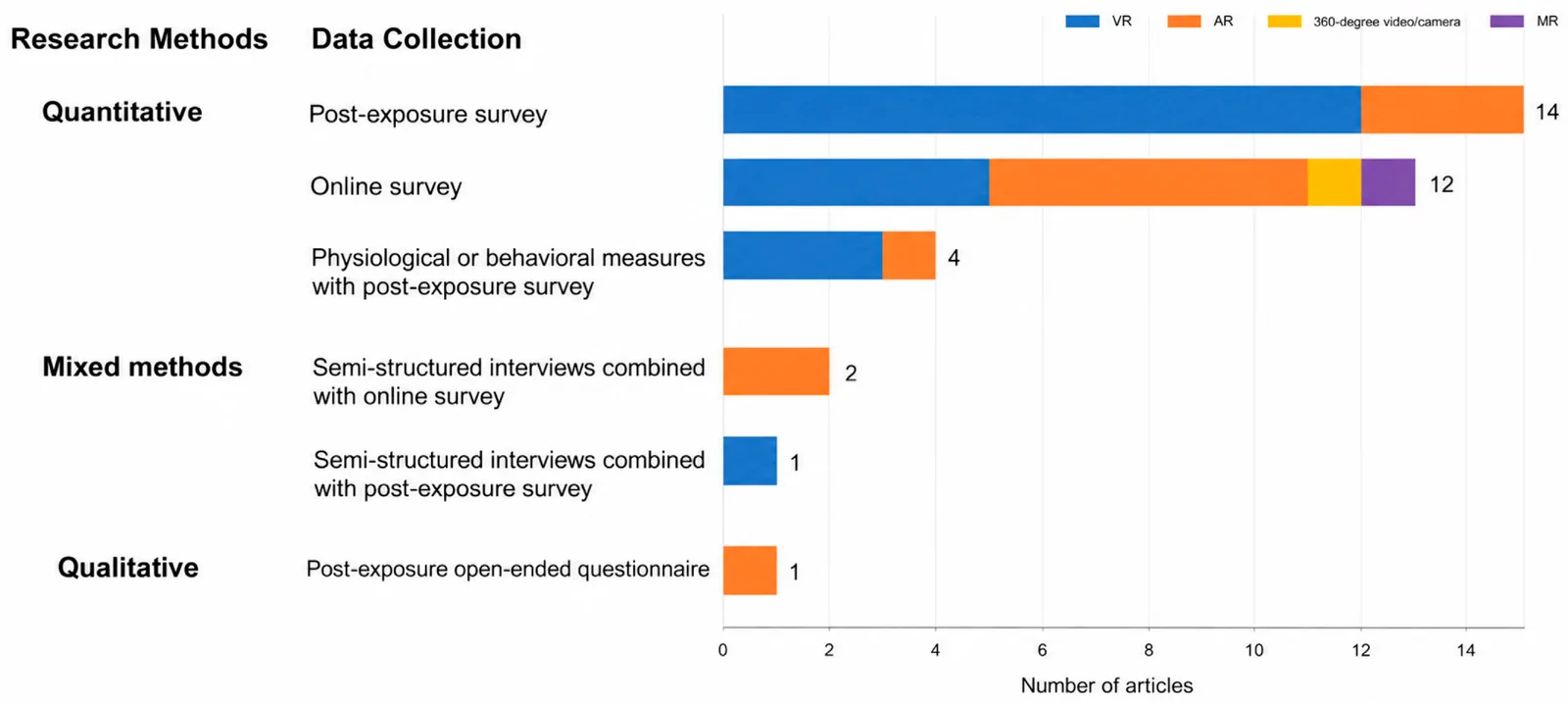
Distribution of the included articles by methodological design.

Figure 5 summarizes the methodological designs used in the articles. Most articles adopted quantitative research designs (*n* = 30, 88.2%), three articles used mixed-methods designs (*n* = 3, 8.8%) and one article employed an exclusively qualitative design (*n* = 1, 2.9%). Among the quantitative articles, 14 used post-exposure surveys, 12 used online surveys, and four combined post-exposure surveys with physiological or behavioral measures. The mixed-methods articles combined semi-structured interviews with either online surveys (*n* = 2) or a post-exposure survey (*n* = 1), while the qualitative article used a post-exposure open-ended questionnaire.

Methodological patterns differed by technology type. VR research was overwhelmingly quantitative: 19 of the 20 VR articles adopted quantitative methods, while one used a mixed-methods design. Post-exposure surveys were particularly common in VR articles (*n* = 11), and three additional VR articles combined post-exposure surveys with physiological or behavioral measures, including eye-tracking, electrodermal or cardiac activity, heart rate, skin conductance, brand-memory measures, and EEG. Five VR articles used online surveys. AR articles displayed greater methodological diversity, comprising nine quantitative articles, two mixed-methods articles, and the only exclusively qualitative study. Online surveys were the most common approach in AR research (*n* = 5), followed by post-exposure surveys (*n* = 3). One AR article combined physiological or behavioral measures with a post-exposure survey, two combined semi-structured interviews with online surveys, and one collected qualitative data through a post-exposure open-ended questionnaire. Only one article examined 360-degree video, and it employed a quantitative online survey. Similarly, the single MR article employed a quantitative online survey. Given that 360-degree video and MR were each represented by only one article, it is difficult to identify stable methodological patterns for these technologies.

Overall, the field is dominated by quantitative research, with post-exposure surveys and online surveys being the most frequently used data collection methods. Qualitative and mixed-methods approaches remain comparatively uncommon, indicating limited methodological diversity in the current evidence base.

### 4.2. Theoretical Mapping

This scoping review identified 36 distinct theories and models were identified across the 34 included articles. As shown in Table 4, several theories were applied across more than one technology type, whereas others were specific to VR, AR, MR, or 360-degree video research. VR articles drew on a broader range of theoretical perspectives than AR articles, with 25 distinct theories or models identified in VR research compared with 15 in AR research. Because MR and 360-degree video were each represented by only one article, no stable theory pattern could be identified for either technology.

#### 4.2.1. Theories Used Across Technology Types

The technology acceptance model (TAM) was the most frequently used model across VR and AR and MR articles (*n* = 7). TAM-based articles examined how perceived usefulness, perceived ease of use, perceived enjoyment, subjective norms, satisfaction, attitudes, and related variables predicted intentions to adopt, use, purchase, recommend, or continue using immersive sport technologies (Burkett et al., 2026; Goebert & Greenhalgh, 2020; Langaro et al., 2022; Lei & Ngan, 2024; Peng & Oh, 2024; Rynarzewska, 2018; Selvaratnam et al., 2026).

Flow theory was used to explain how immersive technological features and media characteristics, such as VR environments, interactivity, vividness, telepresence, and sport involvement, facilitate spectators’ optimal psychological experiences during sport viewing. Across these studies, flow experience primarily functioned as a central psychological mechanism linking immersive technology attributes to spectator outcomes, including enjoyment, satisfaction, engagement, viewing experience, and subsequent behavioral intentions (Chang et al., 2024; D. Kim & Ko, 2019; Kunz & Santomier, 2020; Oh et al., 2022; Rogers et al., 2019; Uhm et al., 2020).

The biased competition model was used to explain how spectators allocate attention when bottom-up stimulus salience and top-down goals compete during immersive sport viewing (Ahn & Ko, 2024; Chang et al., 2024; Y. Kim & Ahn, 2026). The elaboration likelihood model was used to explain how involvement and message processing routes influence spectators’ responses to immersive sport media (Goebert et al., 2022; D. Kim & Ko, 2019). The stimulus–organism–response framework was used across VR and AR articles to explain how immersive media features and viewing conditions function as environmental stimuli that influence spectators’ internal cognitive and affective states, which subsequently shape behavioral responses (Chung et al., 2025; Y. Kim & Ahn, 2026). The limited-capacity model of motivated mediated message processing (LC4MP) appeared in VR and 360-degree video articles and was used to explain how immersive media may compete for limited cognitive resources, influencing attention, memory, and potential overload (Jang et al., 2022; Walton, 2025). The single MR article additionally incorporated Social Identity Theory alongside TAM to examine how dimensions of sport-fan identification relate to technology evaluations and purchase intention (Selvaratnam et al., 2026).

#### 4.2.2. Theories Used in VR

VR research was characterized by a combination of experiential, cognitive, affective, and technology-adoption theories. Telepresence theory was applied in four articles (D. Kim & Ko, 2019; Oh et al., 2022; Petit et al., 2023; Walton, 2025), whereas presence theory was used in one article (Uhm et al., 2020). Together with flow theory, these theoretical perspectives were used to explain immersive experiences, perceived proximity, and satisfaction in VR sport spectatorship. Dynamic human-centered communication systems theory was used to explain real-time cognitive and emotional processing during VR-mediated sport viewing, including attention, arousal, and memory-related outcomes (Lee & Zhou, 2025; Paek et al., 2025).

In addition to TAM, VR studies used UTAUT2, TAM3, the theory of planned behavior, uses and gratifications theory, diffusion of innovation theory, the integrated technology adoption paradigm, and expectancy value theory to explain adoption, use intention, user segmentation, and spectator motivation (Capasa et al., 2022; Kang, 2020; Kunz & Santomier, 2020; Park et al., 2025; Peng & Oh, 2024; Rynarzewska, 2018). Construal level theory was applied to examine psychological distance and VR adoption (Capasa et al., 2022). Grounded cognition theory, attachment theory, and image transfer theory were jointly applied to explain how VR-mediated experiences could influence psychological distance, team attachment, and sponsor attachment (Petit et al., 2023).

Other theories, such as affective expectation theory, emotion dynamics framework, and poison theory, were jointly used to explain affective expectations, emotional fluctuations, and the potentially adverse effects of immersive sport-viewing experiences (Chang et al., 2024). Hedonic adaptation theory and satiation theory were used to explain how prolonged exposure may reduce positive VR viewing experiences and how interruptions may mitigate such declines (Oh et al., 2022).

#### 4.2.3. Theories Used in AR

AR research used theories related to experience design, technology acceptance, motivation, resistance, attention, emotional responses, consumer innovativeness, and sensory adaptation. Experience economy theory and experiential marketing theory were used to explain how AR can generate entertainment, educational, aesthetic, escapist, and experience value-based responses (Du et al., 2024; Lei & Ngan, 2024; Ngan & Lei, 2025). The technology readiness and acceptance model extended TAM by incorporating individual readiness factors such as optimism and innovativeness (Goebert & Greenhalgh, 2020). The motivated consumer innovativeness framework was used to explain how consumers’ intrinsic motivation to adopt novel technologies influences their acceptance of AR-enhanced sport experiences and their willingness to engage with innovative viewing services (Y. Kim & Ahn, 2026; S. Kim & Manoli, 2024). Innovation resistance theory was used to examine postponement, rejection, and opposition to AR technologies in stadium contexts (Uhlendorf & Uhrich, 2024).

Self-determination theory was applied to examine how AR may support or undermine spectators’ autonomy, competence, and relatedness during sport viewing (Rogers et al., 2017, 2019). The pleasure–arousal model was used to explain spectators’ affective reactions to AR-enhanced sport content, whereas selective attention theory was applied to examine how AR elements direct or compete for spectators’ attentional resources (Y. Kim & Ahn, 2026). Dual-process theory of habituation and sensitization, habituation theory, and sensory information capacity theory were used to explain sensitization, habituation, repeated-exposure and viewing duration effects, irritation, and related psychological responses to AR-enhanced sport content (Ahn & Ko, 2024; Y. Kim & Ahn, 2026).

### 4.3. Thematic and Variable Mapping

The 34 included articles were grouped into two primary themes according to their main research purpose and variable structure: (1) adoption or resistance to immersive technologies and applications of TAMs, and (2) immersive technology experience and psychological response mechanisms. As shown in Table 5, 11 articles focused primarily on adoption or resistance, whereas 23 articles focused primarily on immersive experiences and psychological mechanisms. VR articles were distributed across both themes, with eight VR articles focusing on adoption or resistance and 12 focusing on experience and psychological mechanisms. AR articles were more concentrated in the second theme, with 10 AR articles focusing on experience and psychological mechanisms and two focusing on adoption or resistance. Only one 360-degree video article was identified, and it belonged to the experience and psychological mechanism theme. The single MR article focused on technology adoption and resistance.

#### 4.3.1. Adoption or Resistance to Immersive Technologies

The first theme includes studies that examined why spectators adopt, intend to use, continue using, postpone, reject, or resist immersive sport-viewing technologies. TAM and its extensions were central to this theme. Perceived usefulness and perceived ease of use appeared as core constructs and were often examined alongside perceived enjoyment, subjective norms, satisfaction, attitude, price value, technology readiness, and facilitating conditions (Capasa et al., 2022; Goebert & Greenhalgh, 2020; Langaro et al., 2022; Peng & Oh, 2024). More recent work further extended TAM by examining flow as an experiential outcome associated with technology acceptance. In the model examined by Burkett et al. (2026), technology innovation was associated with perceived usefulness and perceived ease of use, which were linked to perceived enjoyment and, subsequently, flow experience.

Several VR studies extended adoption models by incorporating sport-specific and user-specific constructs. For example, studies examined information consumption, social interaction, interest curiosity, self-construal, innovativeness, habit, hedonic motivation, technology fluidity, computer ownership, VR tool ownership, audience factors, use factors, social factors, and system factors as antecedents of VR adoption, use intention, purchase intention, adoption beliefs, adoption attitudes, and behavioral intention (Capasa et al., 2022; Kang, 2020; Kunz & Santomier, 2020; Langaro et al., 2022; Rynarzewska, 2018). Other studies moved beyond a simple adopter/non-adopter distinction by categorizing users into continuers, discontinuers, potential adopters, and resistors, thereby addressing slower diffusion patterns and pro-innovation bias in immersive sport technology research (Park et al., 2025).

Resistance-oriented research was less common but important. In the AR context, Uhlendorf and Uhrich (2024) examined how distraction from the live experience, interference with fan rituals supporting the team, reduced social interactions, reduced emotionality in discussions, risk of personal image damage, loss of stadium atmosphere, and fan identity incongruence predicted postponement, rejection, and opposition. This line of work suggests that immersive technology adoption should not be interpreted only as a positive acceptance process; rather, spectators may simultaneously perceive benefits and experiential, social, tradition-related, and image-related barriers when evaluating whether to use AR or VR in sport-viewing contexts (Table 6).

#### 4.3.2. Immersive Technology Experience and Psychological Response

The second theme includes studies that examined how immersive technologies shape spectators’ experiential, cognitive, affective, evaluative, and behavioral responses. Across the included studies, antecedent variables were grouped into four broad categories: technology type, technology features, sensory imagery, and user motivation.

Technology type variables were usually operationalized as experimental or comparative conditions, such as traditional broadcast versus coach-mode or mascot-mode broadcast, 2D versus VR viewing, AR versus non-AR viewing, actual AR versus indirect AR, uninterrupted 2D-screen viewing versus interrupted VR viewing, static VR broadcast versus visualization-enhanced VR broadcast versus fully integrated VR broadcast, high-tech versus non-high-tech cameras, and Google Glass-based viewing. These comparisons allowed researchers to examine whether different immersive formats, visualization designs, camera configurations, and modes of technological integration produce different spectator responses (Ahn & Ko, 2024; Frewen & Vincent, 2024; Goebert et al., 2022; Jang et al., 2022; D. Kim & Ko, 2019; Y. Kim & Ahn, 2026; Lee & Zhou, 2025; Lo et al., 2022; Oh et al., 2022; Paek et al., 2025; Rogers et al., 2017; Vincent & Frewen, 2023; Walton, 2025; Yao et al., 2026). In terms of delivery interfaces, immersive content in the reviewed studies was operationalized through standalone and tethered HMDs (e.g., Oculus Go, Oculus/Meta Quest, Oculus Rift, PlayStation VR, HTC VIVE, and Samsung Gear VR), smartphone-based VR viewers, smartphone AR applications, smart glasses (e.g., Google Glass), and conventional 2D screens used as comparison conditions. The device and interface employed in each study are detailed in Appendix A (Table A1).

Technology feature variables included novelty, vividness, informativeness, and interactivity. Novelty refers to the perceived newness or originality of an immersive sport-viewing experience; vividness refers to the sensory richness and representational quality of the medium; informativeness refers to the extent to which the medium provides useful and relevant sport-related information; and interactivity refers to users’ perceived ability to control, respond to, or modify aspects of the media experience (Du et al., 2024; Jang et al., 2022; D. Kim & Ko, 2019; Lei & Ngan, 2024).

Sensory imagery was examined as a cognitive antecedent through visual and aural imagery, indicating how spectators mentally simulate sensory aspects of a forthcoming or mediated VR experience (Chung et al., 2025). Motivated consumer innovativeness was examined as a user-level antecedent, including functional, hedonic, cognitive, and social motivations for engaging with innovative sport-viewing technologies (S. Kim & Manoli, 2024) (Table 7).

##### Experience-Related Variables

Table 8 summarizes experience-related variables identified in the studies. Presence was one of the most frequently examined immersive experience constructs. It was conceptualized as spectators’ sense of being in a mediated sport environment and, in some studies, was further divided into spatial, temporal, and social presence (Chung et al., 2025; Frewen & Vincent, 2024; Jang et al., 2022; Lee & Zhou, 2025; Lo et al., 2022; Uhm et al., 2020; Vincent & Frewen, 2023). Telepresence was closely related but more specifically referred to the feeling of being transported to a remote sport environment through mediated technology (D. Kim & Ko, 2019; Oh et al., 2022; Walton, 2025). Immersion was additionally examined as a broader subjective state of being deeply enveloped in the mediated sport-viewing environment (Yao et al., 2026). Flow experience was also central to VRS research. Flow was generally described as a state of deep involvement and absorption during sport viewing, and it was often linked to enjoyment, cognitive absorption, time distortion, and satisfaction (Chang et al., 2024; D. Kim & Ko, 2019; Oh et al., 2022).

Beyond immersion-related constructs, the reviewed studies also assessed the quality of spectators’ technology-mediated experiences. Comfort captured the extent to which spectators experienced the viewing technology as physically and psychologically comfortable (Yao et al., 2026), whereas user experience reflected a broader evaluation of the usability and experiential quality of the visualization system (Lo et al., 2022).

AR studies also examined multidimensional experiences, including aesthetic, educational, entertainment, and escapist experiences, as pathways through which AR features influence rewatching, recommendation, and other behavioral intentions (Du et al., 2024). Engagement was conceptualized as a multidimensional construct involving cognitive, affective, physical, and novelty-related engagement, and was linked to subsequent behavioral responses (Y. Kim & Ahn, 2026; Lei & Ngan, 2024; Ngan & Lei, 2025; Yao et al., 2026).

##### Psychological Response Variables

Table 9 summarizes psychological response variables. Cognitive variables included cognitive resource allocation, visual attention, cognitive absorption, memory, and stimulus perception. Cognitive resource allocation was examined using psychophysiological indicators such as heart rate-related measures, while visual attention was measured through eye-tracking indicators such as fixation count and fixation duration (Lee & Zhou, 2025; Paek et al., 2025). Cognitive absorption reflected spectators’ deep cognitive involvement and concentration during immersive sport viewing (Ahn & Ko, 2024). Memory outcomes included brand recall, brand recognition, and recognition sensitivity, reflecting the extent to which spectators encoded and retained information embedded in immersive sport-viewing environments (Lee & Zhou, 2025; Paek et al., 2025; Walton, 2025). Stimulus perception included perceived visual and aural stimuli, which were examined as sensory inputs that may enhance presence and consumption intentions (Chung et al., 2025). Comprehension-related outcomes included general comprehension, spatial understanding, and game understanding. These variables reflected spectators’ ability to interpret presented information, understand the spatial relationships between visualized data and the sporting environment, and follow game events and statistics during immersive or augmented sport viewing (Lo et al., 2022; Yao et al., 2026). Workload captured the cognitive, physical, temporal, and effort-related demands experienced while using situated sport visualizations (Lo et al., 2022).

Affective variables included enjoyment, arousal, and emotion dynamics. Enjoyment reflected the pleasure or entertainment value associated with immersive sport viewing (Ahn & Ko, 2024; Jang et al., 2022; Rogers et al., 2017). Arousal was examined as spectators’ level of activation or excitement during immersive sport experiences and was measured through self-report or physiological indicators (Chang et al., 2024; Chung et al., 2025; Lee & Zhou, 2025; Uhm et al., 2020). Emotion dynamics captured changes in emotional intensity or valence across different moments of the viewing experience (Chang et al., 2024).

Satisfaction and attitude appeared as evaluative psychological responses. Satisfaction referred to spectators’ overall evaluation of immersive sport-viewing experiences (Chang et al., 2024; Frewen & Vincent, 2024; D. Kim & Ko, 2019; S. Kim & Manoli, 2024; Lo et al., 2022; Oh et al., 2022; Vincent & Frewen, 2023). Attitude captured positive or negative evaluations of the technology, the viewing experience, or the sport object itself and included both hedonic and utilitarian evaluations (Goebert et al., 2022; Lei & Ngan, 2024; Uhm et al., 2020). Psychological need variables, including relatedness, autonomy, and competence, were examined in relation to spectators’ enjoyment and motivational experience during AR-enhanced sport viewing (Rogers et al., 2017).

##### Negative Experience Variables

Many studies emphasized positive experiential outcomes, while other studies examined negative responses to immersive technologies. As shown in Table 10, negative variables included irritation, distraction, and cognitive load. Irritation referred to negative affect or discomfort caused by excessive information, intrusive visual elements, interface complexity, or overstimulation during immersive sport viewing (Ahn & Ko, 2024). Distraction referred to the diversion of spectators’ attention from core sport content due to competing visual or technological stimuli (Jang et al., 2022). Cognitive load referred to the mental effort required to process information-rich immersive environments and was examined as a potential constraint on spectator enjoyment (Rogers et al., 2017).

These findings show that immersive technologies may be associated with both facilitating and inhibiting responses. They were examined in relation to presence, enjoyment, and engagement, but they may also introduce attentional competition, processing burden, and negative affective responses. This dual effect is important for understanding immersive sport spectatorship as a behavioral phenomenon rather than simply a technological enhancement.

##### Behavioral Intention Variables

Table 11 summarizes behavioral intention variables examined in the included articles. To further distinguish these outcomes, the identified variables were reorganized into four categories according to the type of intended behavior: media and content engagement intentions, consumption intentions, advocacy and communication intentions, and revisit intentions. Most of these variables reflect self-reported intentions or anticipated future responses rather than directly observed behavior.

Media and content engagement intentions included intention to follow social media, intention to watch again, stickiness, and media engagement intention. Intention to follow social media was examined as a form of continued engagement with sport-related media content (Jang et al., 2022), while intention to watch again was examined in relation to spectators’ willingness to re-engage with immersive sport content (Du et al., 2024; Goebert et al., 2022). Stickiness captured spectators’ willingness to remain connected to and continue engaging with immersive sport content (Ahn & Ko, 2024), whereas media engagement intention reflected intentions to prolong viewing, increase viewing frequency, and repeatedly return to AR-enhanced sport broadcasting (Y. Kim & Ahn, 2026). Consumption intentions comprised consumption intention and intention to pay. Consumption intention was examined in relation to sensory experience and VR sport spectatorship (Chung et al., 2025), whereas intention to pay was examined in AR sport video contexts (Du et al., 2024).

Advocacy and communication intentions included intention to recommend and word of mouth. Intention to recommend was examined in AR sport video contexts (Du et al., 2024). Word of mouth was examined in both positive and negative forms (Goebert et al., 2022; S. Kim & Manoli, 2024; Lei & Ngan, 2024). Revisit intentions comprised revisit intention and future event visiting intention. Revisit intention was examined in relation to AR-enhanced sport event experiences (Ngan & Lei, 2025), while future event visiting intention was examined as a subsequent response to AR-enhanced mega-sport event experiences (Lei & Ngan, 2024). Overall, the evidence is concentrated on intended future responses across engagement, consumption, advocacy, and revisit domains, with comparatively limited evidence based on directly observed spectator behavior.

##### Moderating Variables

Table 12 summarizes the moderating variables. Moderating variables were identified primarily in relation to individual characteristics and sport-context factors. Individual-level moderators included sport involvement, hedonic innovativeness, past VR experience, and need for cognition. Sport involvement was the most frequently examined moderator and was used to explain why spectators with different levels of sport involvement responded differently to immersive viewing technologies (Ahn & Ko, 2024; Goebert et al., 2022; Jang et al., 2022; D. Kim & Ko, 2019; Y. Kim & Ahn, 2026). Hedonic innovativeness reflected spectators’ tendency to seek novel and enjoyable technology-mediated experiences, which may condition how playful AR features influence their engagement during immersive sport viewing (Y. Kim & Ahn, 2026). Past VR experience reflected spectators’ prior familiarity with VR technology, which may condition the extent to which immersive VR environments facilitate flow experiences during sport viewing (Chang et al., 2024). Need for cognition reflected spectators’ tendency to engage in and enjoy effortful thinking, which may condition how different information-seeking technologies influence their responses during sport viewing (Rogers et al., 2017).

Sport-context moderators included game situation and game rivalry. Game situation, operationalized as a close versus decisive game, was used to assess whether the effects of immersive VR on spectators’ psychological responses differed according to the competitive uncertainty of the event (Chang et al., 2024). Game rivalry was examined as a contextual factor that could strengthen or weaken spectators’ experiential and evaluative responses to immersive sport viewing (D. Kim & Ko, 2019).

These findings suggest that reported relationships involving immersive technologies may vary according to individual differences among spectators and the competitive context in which the sport experience occurs.

##### Control Variables

Control variables were explicitly reported in 13 of the 34 included articles. The most frequently reported controls were sport-related involvement and identification variables, including sport or viewing involvement, team identification, and fan identification (Ahn & Ko, 2024; Chang et al., 2024; Chung et al., 2025; Goebert et al., 2022; Jang et al., 2022; Y. Kim & Ahn, 2026; Lee & Zhou, 2025; Oh et al., 2022; Paek et al., 2025; Walton, 2025). Other controls included prior viewing or technology-related characteristics, such as past game-watching experience, prior AR experience, technology readiness, media use, and preference for new technology (Ahn & Ko, 2024; Kang, 2020; Rogers et al., 2017). Demographic characteristics, including gender, age, income, education, and race, were also controlled in some articles (Kang, 2020; Rogers et al., 2017). A smaller number of articles controlled for study-specific factors such as emotion reactivity and game satisfaction (Chang et al., 2024; S. Kim & Manoli, 2024). Overall, the use of control variables was heterogeneous across the literature, with many articles not explicitly reporting control variables. Detailed article-level information is provided in Appendix A (Table A3).

#### 4.3.3. Proposed Integrative Conceptual Model in Immersive Sport Spectatorship

The reviewed studies suggest an emerging, fragmented evidence structure. Technology type and technology features were examined as antecedents; experiential variables such as presence, telepresence, flow, and engagement were frequently examined as experiential and psychological responses; cognitive and affective responses were also examined in relation to spectators’ evaluations; and behavioral intentions represent the most common outcome category. Negative responses, including irritation, distraction, and cognitive load, are potential barriers that may weaken the positive effects of immersive sport-viewing technologies. In addition, the relationships between technology types or features and psychological responses may be conditioned by individual characteristics (e.g., sport involvement, hedonic innovativeness, past VR experience, and need for cognition) and sport-context characteristics (e.g., game situation and game rivalry).

**Figure 6:**
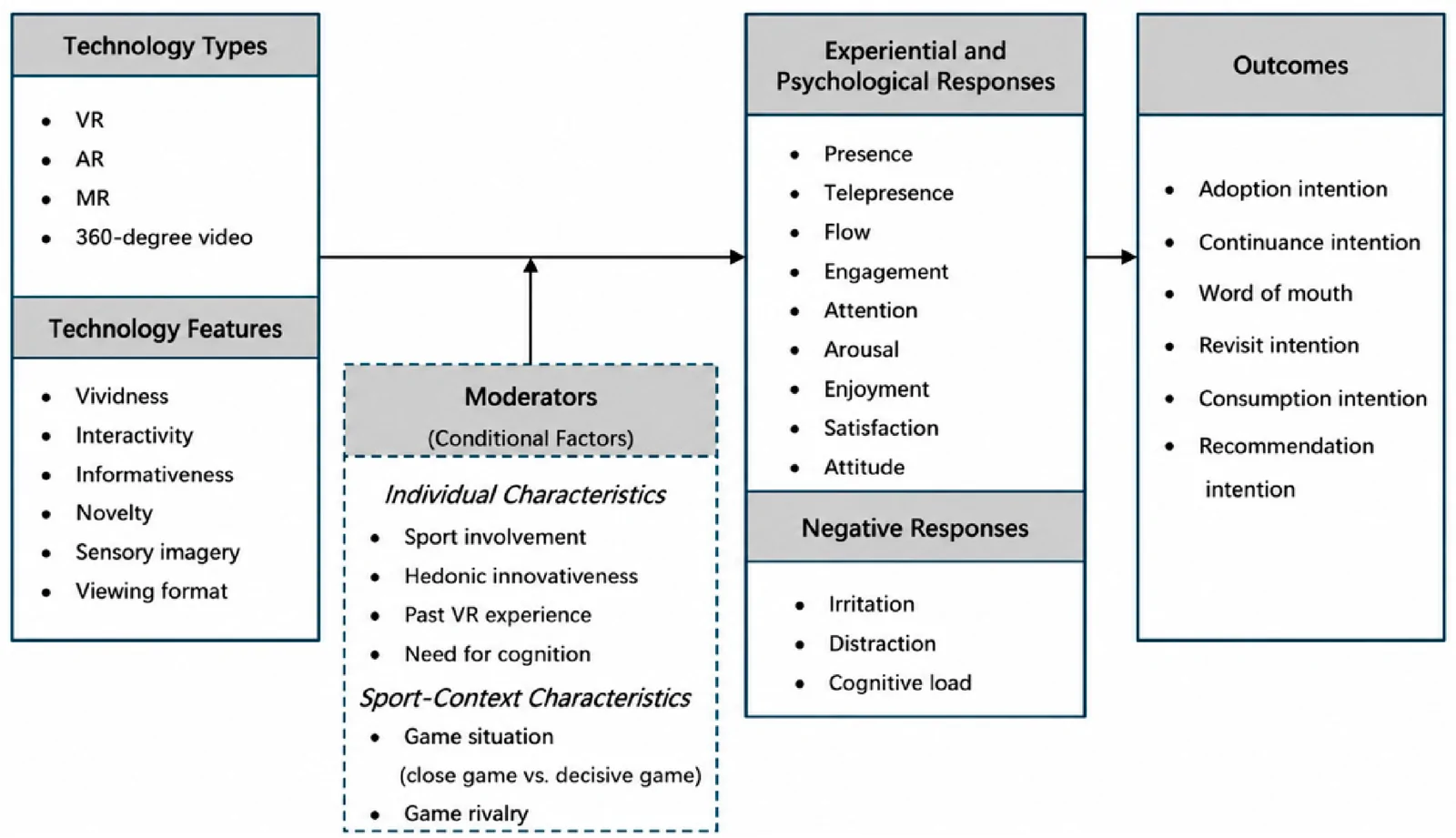
Proposed integrative conceptual model in immersive sport spectatorship.

As illustrated in Figure 6, this proposed integrative conceptual model can be summarized as follows: Immersive technology types and features, including vividness, interactivity, informativeness, novelty, sensory imagery, and viewing format, have been examined in relation to experiential and psychological responses, including presence, telepresence, flow, engagement, attention, arousal, enjoyment, satisfaction, and attitude, while also potentially eliciting negative responses such as irritation, distraction, and cognitive load. These relationships are further conditioned by individual and sport-context characteristics. These experiential and psychological responses were also examined in relation to outcomes such as adoption intention, continuance intention, word of mouth, revisit intention, recommendation intention, and consumption intention.

## 5. Future Research Agenda

The findings of this scoping review suggest that future research on immersive sport spectatorship should move from technology-specific and adoption-oriented studies toward more integrated behavioral explanations (Table 13). Although existing studies have provided important evidence on VR and AR, whereas evidence on MR and 360-degree video remains particularly limited, and the field remains unevenly developed across technologies, theories, methods, samples, sport contexts, and outcome variables. Building on the descriptive, theoretical, thematic, and variable mapping presented in this review, this section identifies four priority directions for future research: conceptual and theoretical integration; comparative and context-sensitive research; methodological and measurement advancement; and the examination of negative responses, boundary conditions, and sustainable use. Rather than asking only whether immersive technologies improve sport-viewing experiences, future research should examine how, why, when, and for whom these technologies influence spectators’ attention, emotion, presence, satisfaction, resistance, and behavioral intentions.

### 5.1. Conceptual and Theoretical Integration

First, future research should clarify the conceptual boundaries of immersive sport spectatorship and integrate the theoretical perspectives used to explain it. The reviewed literature shows that concepts such as immersion, presence, telepresence, flow, engagement, enjoyment, and cognitive absorption are sometimes used in overlapping ways. Future studies should distinguish more clearly between technological immersion and psychological responses to immersion. Technological immersion refers to system-level affordances such as sensory richness, interactivity, spatial perspective, field of view, environmental substitution, and information augmentation. By contrast, presence, flow, engagement, enjoyment, satisfaction, and cognitive absorption refer to spectators’ subjective responses to those affordances.

Clearer conceptual boundaries are also needed between immersive sport spectatorship and adjacent domains such as athlete training, sport education, rehabilitation, performance analysis, officiating, and technical system development. For this field to develop cumulatively, studies should specify whether spectators, fans, viewers, audiences, or consumers are the focal users and whether the outcomes concern viewing experience, psychological response, or sport consumption behavior.

Theoretical integration is equally important. TAM and its extensions have been useful for explaining adoption and use intention, but they do not fully capture the experiential, cognitive, affective, and resistance-related processes involved in immersive sport spectatorship. Future research should therefore integrate acceptance-oriented and resistance-oriented theories within a dual-path framework, rather than treating acceptance and resistance as opposite ends of a single process. Such a framework would allow researchers to examine both adoption and resistance within the same model. It would also help explain how these two processes coexist and shape spectators’ subsequent responses.

Behavioral reasoning theory may provide a useful overarching perspective for this integration because it conceptualizes reasons for adoption and reasons for resistance as distinct but coexisting drivers of behavior. This perspective is particularly relevant to immersive sport spectatorship, where spectators may simultaneously perceive both benefits and barriers associated with immersive technologies (Claudy et al., 2015; Uhrich, 2022).

### 5.2. Comparative and Context-Sensitive Research

Second, future research should conduct more comparative and context-sensitive studies. The current literature is unevenly distributed across immersive technologies: VR has been studied most consistently, AR research has grown more noticeably in recent years, and MR and 360-degree video remains underrepresented. These technologies produce immersion through different mechanisms, which is why findings from one technology should not be automatically generalized to another. VR may intensify presence and telepresence by reconstructing the viewing environment; AR may enhance information access and engagement by augmenting live or mediated sport settings; however, the evidence concerning 360-degree video is currently too limited to characterize its effects with confidence. Conceptually, 360-degree video provides expanded spatial perspective and viewpoint control, but these potential experiential effects require further empirical examination in sport spectatorship contexts.

More direct comparisons are needed across traditional broadcast viewing, AR-enhanced viewing, 360-degree video, and VR viewing under comparable sport-viewing conditions. Such comparisons are particularly important for 360-degree video because the current evidence base is too limited to support stable technology-level conclusions. Such designs would help determine whether spectator responses are driven by the technology category itself; by specific affordances such as vividness, interactivity, informativeness, novelty, or spatial perspective; or by short-term novelty effects associated with emerging media.

Future studies should also broaden the examined contexts and populations. Existing studies are concentrated in a limited number of countries and rely heavily on college students and general population samples. To improve generalizability, future research should examine different spectator groups, including highly identified fans, casual viewers, older adults, families, disabled spectators, international sport tourists, streaming platform users, and long-term users of immersive sport-viewing systems. Studies should also compare different sport types, including team sports, individual sports, esports, para-sports, motorsports, combat sports, and mega-events. Cross-cultural research would be particularly valuable because spectators’ responses to immersive technologies may vary according to sport traditions, media habits, social viewing norms, privacy expectations, and technology readiness.

### 5.3. Methodological and Measurement Advancement

Third, future research should expand methodological diversity and improve measurement rigor. The reviewed literature is dominated by quantitative and experimental designs, with limited use of qualitative, longitudinal, field-based, and mixed-methods approaches. Although experiments and surveys are useful for testing relationships among technology features, psychological mechanisms, and behavioral intentions, they may not fully capture how spectators interpret, negotiate, and sustain immersive sport-viewing experiences in naturalistic settings.

Qualitative methods such as interviews, focus groups, diary studies, think-aloud protocols, ethnography, and user-experience observation could provide richer insight into why spectators find certain immersive features meaningful, distracting, enjoyable, confusing, or unnecessary. Longitudinal and field-based designs are also needed because many existing studies rely on one-time exposure or intention-based outcomes. Spectators’ responses may change after repeated use; novelty may decline, discomfort may become more salient, or users may develop new viewing habits over time.

Future studies should also use more multimodal measurement. Several reviewed studies have already incorporated psychophysiological or attention-based indicators, including heart rate-related measures, eye tracking, visual attention, arousal, and memory-related outcomes (Lee & Zhou, 2025; Paek et al., 2025; Walton, 2025). Building on this work, future research should combine self-report data with behavioral logs, interaction data, physiological indicators, eye-tracking measures, and actual post-viewing behaviors. This would allow researchers to examine not only what spectators report experiencing, but also how they attend, react, and behave during immersive sport viewing.

### 5.4. Negative Responses, Boundary Conditions, and Sustainable Use

Fourth, future research should examine negative responses, boundary conditions, and sustainable use more systematically. Much of the existing literature emphasizes positive outcomes such as presence, flow, enjoyment, engagement, satisfaction, and behavioral intention. However, immersive technologies may also produce irritation, distraction, cognitive load, discomfort, resistance, and discontinuance (Ahn & Ko, 2024; Jang et al., 2022; Rogers et al., 2017; Uhlendorf & Uhrich, 2024). These negative responses are not peripheral issues; rather, they are central to understanding whether immersive sport technologies can generate sustainable spectator engagement. Future research should therefore examine positive and negative responses within the same study to determine when they coexist and when improvements in one dimension of the immersive experience produce trade-offs in another.

Future research should identify when immersive features enhance sport spectatorship and when they interfere with the core viewing experience. AR overlays may improve comprehension when they provide timely and relevant information, but they may also distract spectators when they are visually intrusive or poorly synchronized with game action. VR may enhance presence and emotional involvement, but it may also create discomfort, usability barriers, or social isolation. 360-degree video may increase viewing autonomy, but it may also require additional attentional effort and increase the risk of missing important game moments. Given the very limited evidence on 360-degree video, future research should examine whether greater viewpoint control enhances viewing autonomy or instead increases attentional demands and the risk of missing important game moments.

Boundary conditions should be examined at individual, technological, and contextual levels. Individual moderators may include sport involvement, fan identification, age, prior technology experience, innovativeness, motion sensitivity, accessibility needs, and motivation. Technological moderators may include device comfort, latency, interface complexity, information density, field of view, interactivity, and visual realism. Contextual moderators may include sport type, viewing location, social setting, event importance, and live versus recorded viewing.

Ultimately, future studies should develop integrated behavioral frameworks that examine relationships among immersive technology types and features, psychological responses, boundary conditions, and behavioral intentions or related outcomes. A cumulative model of immersive sport spectatorship should test how technology features such as vividness, interactivity, informativeness, novelty, spatial perspective, sensory imagery, and environmental substitution are associated with responses such as presence, flow, engagement, attention, arousal, satisfaction, cognitive load, and irritation. It should also examine how these responses relate to outcomes such as adoption intention, continuance intention, word of mouth, revisit intention, intention to pay, consumption intention, resistance, and discontinuance.

## 6. Discussion

This scoping review mapped empirical research on immersive technologies in sport spectatorship, focusing on how VR, AR, MR and 360-degree video have been examined in relation to spectators’ experiential and psychological responses, behavioral intentions, and related outcomes. The findings show that immersive sport spectatorship is an emerging but unevenly developed research domain. Among the 34 included articles, comprising 40 independent studies, VR was the most frequently examined technology, followed by AR, whereas 360-degree video remained substantially underrepresented. The literature was also concentrated in a limited number of national contexts, relied heavily on university student and general population samples, and was dominated by quantitative designs relying primarily on post-exposure and online surveys.

A central insight of this review is that immersive technologies should not be understood merely as advanced viewing devices or sport marketing tools. In sport spectatorship, VR, AR, MR, and 360-degree video have been examined as mediated viewing environments in relation to spectators’ attention, sensory processing, emotional engagement, evaluative judgments, and behavioral intentions. Across the reviewed articles, technology-related features such as vividness, interactivity, informativeness, novelty, sensory imagery, spatial perspective, and viewing mode were examined in relation to responses such as presence, telepresence, flow, engagement, attention, arousal, enjoyment, satisfaction, and attitude. These responses were also examined in relation to behavioral intentions and related outcomes such as adoption intention, continuance intention, word of mouth, revisit intention, recommendation intention, consumption intention, and intention to pay (Chung et al., 2025; Du et al., 2024; D. Kim & Ko, 2019; Lei & Ngan, 2024; Ngan & Lei, 2025).

The findings also reveal the dual nature of immersive sport spectatorship. Although immersive technologies can enhance presence, enjoyment, engagement, satisfaction, and behavioral intention, they may also produce irritation, distraction, cognitive load, discomfort, resistance, and discontinuance (Ahn & Ko, 2024; Jang et al., 2022; Rogers et al., 2017; Uhlendorf & Uhrich, 2024). These positive and negative responses should not necessarily be treated as opposite ends of a single continuum. Rather, the reviewed evidence suggests two possible relationships between them: coexistence and trade-off. Coexistence occurs when positive and negative responses represent different dimensions of the same viewing experience and are experienced simultaneously without one necessarily eliminating the other. For example, Rogers et al. (2019) showed that Google Glass could provide novelty while also generating distraction and discomfort.

By contrast, a trade-off occurs when an improvement in one aspect of the immersive experience is accompanied by deterioration in another outcome. For example, Lee and Zhou (2025) found that VR increased presence, arousal, and attention but reduced recognition of in-stadium sponsor signage, while Walton (2025) reported that VR increased telepresence but reduced brand recall relative to 2D viewing. These findings indicate that stronger immersion or attention to immersive content may compete with the processing of other information.

Accordingly, positive responses such as novelty, presence, and telepresence may coexist with distraction or discomfort, whereas clearer conflicts emerge when enhanced immersion or attentional engagement is accompanied by poorer processing or memory for other viewing information. However, because relatively few studies have examined positive and negative responses simultaneously, the current evidence does not support a definitive classification of all response pairs as either coexisting or conflicting.

This duality is particularly important in sport settings, where spectators must continuously follow dynamic game action while processing contextual information, emotional cues, social meanings, and commercial messages. The behavioral value of immersive technologies therefore depends not on technological novelty alone, but also on how well immersive features support spectators’ viewing goals without overloading or distracting them.

The findings further indicate that the effects of immersive sport-viewing technologies are conditional rather than uniform across spectators and viewing contexts. Several studies identified individual characteristics, including sport involvement, hedonic innovativeness, past VR experience, and need for cognition, as factors that may strengthen, weaken, or alter spectators’ responses to immersive technologies (Ahn & Ko, 2024; Goebert et al., 2022; Jang et al., 2022; D. Kim & Ko, 2019; Y. Kim & Ahn, 2026; Chang et al., 2024; Rogers et al., 2017). Sport-context characteristics, such as game situation and game rivalry, were also shown to shape experiential and evaluative outcomes (D. Kim & Ko, 2019; Chang et al., 2024). These findings suggest that the same immersive affordance may produce different psychological and behavioral consequences depending on who the spectator is and under what competitive conditions the viewing experience occurs. Immersive sport spectatorship should therefore be understood not as a universally beneficial technological intervention, but as a context-dependent process in which individual predispositions and sport-specific conditions influence the relationships between technology affordances, psychological mechanisms, and behavioral outcomes.

### 6.1. Theoretical Contributions

This review makes three theoretical contributions. First, it clarifies the conceptual boundary of immersive sport spectatorship by focusing on VR, AR, MR and 360-degree video in spectator, fan, viewer, audience, and consumer contexts. This distinction separates immersive sport spectatorship from adjacent domains such as athlete training, sport education, rehabilitation, performance analysis, officiating, and technical system development.

Second, the review distinguishes technological immersion from spectators’ psychological responses to immersion. Technological immersion refers to system-level affordances such as sensory richness, interactivity, spatial perspective, environmental substitution, and information augmentation. By contrast, presence, telepresence, flow, engagement, enjoyment, satisfaction, and cognitive absorption refer to spectators’ subjective responses to those affordances. This distinction helps avoid the assumption that more technologically immersive systems automatically produce better spectator experiences.

Third, the review integrates a fragmented theoretical landscape. TAM and related acceptance models are useful for explaining adoption and use intention, but they do not fully explain the experiential, cognitive, affective, and resistance-related dimensions of immersive sport spectatorship. The reviewed literature suggests that theories of presence, flow, telepresence, LC4MP, biased competition, self-determination, experience economy, innovation resistance, and dynamic information processing should be connected to explain immersive sport spectatorship as a multistage behavioral process involving technology perception, psychological experience, evaluative response, and behavioral consequence.

### 6.2. Practical Implications

The findings offer practical implications for sport organizations, broadcasters, streaming platforms, event managers, sponsors, and immersive technology developers. First, practitioners should design immersive sport-viewing experiences around spectators’ psychological needs rather than technological novelty alone. Immersive features are valuable only when they help spectators understand the sport, feel closer to the event, enjoy the experience, interact meaningfully with content, or sustain engagement.

Second, practitioners should balance information enrichment with attentional manageability. AR overlays, real-time statistics, tactical graphics, multi-angle replays, virtual viewpoints, and interactive interfaces can improve spectators’ comprehension and enjoyment when they are relevant and well timed. However, excessive, intrusive, or poorly synchronized information may produce irritation, distraction, or fatigue in spectators. Designers should therefore prioritize clarity, timing, optionality, and contextual relevance.

Third, immersive sport experiences should be tailored to different spectator segments and evaluated with both positive and negative user-experience indicators. Highly involved fans may value tactical information and advanced camera control, whereas casual viewers may prefer simplified guidance, entertainment-oriented features, or social interaction. Adoption intention, intention to pay, word of mouth, revisit intention, and continuance intention should be assessed alongside irritation, distraction, cognitive load, discomfort, resistance, and discontinuance.

### 6.3. Limitations

This review has several limitations. First, the search was limited to English-language peer-reviewed journal articles found on Scopus, Web of Science, and ProQuest only. Relevant work published in other languages, conference proceedings, dissertations, books, technical reports, and industry reports may therefore have been excluded.

Second, although the search strategy incorporated a relatively broad range of immersive-technology terms, including “virtual reality,” “augmented reality,” “360-degree,” “extended reality,” “XR,” “mixed reality,” “MR,” “immersive media,” “immersive video,” and “spatial computing,” the terminology used to describe emerging immersive technologies continues to evolve. Studies using alternative or newly emerging terminology may therefore not have been captured by the search strategy.

Another important limitation of the current evidence base is its strong reliance on self-reported behavioral intentions rather than directly observed behavior. Although prior studies have examined intentions to use, revisit, recommend, pay, continue viewing, or engage with immersive sport media, comparatively little evidence documents whether these intentions translate into actual spectator behavior over time. Future research should therefore incorporate behavioral logs, repeated-use data, actual purchasing or attendance behavior, and longitudinal measures to examine the intention–behavior relationship in immersive sport spectatorship.

Lastly, the evidence base remains small and heterogeneous. Thirty-four empirical journal articles, comprising 40 independent studies, met the inclusion criteria, and most focused on VR or AR. MR and 360-degree video were each represented by only one article. The frequent use of university student and general population samples; and the diversity of technologies, sport contexts, theories, and methods limit direct comparison and generalizability. In addition, the search was completed on 10 July 2026, so studies published after that date were not included.

## 7. Conclusions

This scoping review examined empirical research on immersive technologies in sport spectatorship, focusing on VR, AR, MR and 360-degree video and their relationships with spectators’ psychological mechanisms and behavioral outcomes. By mapping 34 empirical articles the review shows that immersive sport spectatorship has gained increasing scholarly attention but remains unevenly developed across technology types, theoretical perspectives, methods, geographical contexts, participant groups, and outcome variables.

The findings suggest that immersive sport spectatorship should be understood as a behavioral phenomenon rather than merely a technological innovation. Immersive technology affordances such as vividness, interactivity, informativeness, novelty, sensory imagery, and spatial perspective can shape psychological mechanisms such as presence, telepresence, flow, engagement, attention, arousal, enjoyment, satisfaction, attitude, cognitive load, and irritation. These mechanisms are linked to behavioral outcomes, including adoption intention, continuance intention, word of mouth, revisit intention, recommendation intention, consumption intention, intention to pay, resistance, and discontinuance. Additionally, the findings suggest that these relationships may vary according to individual characteristics and sport-context conditions, highlighting the conditional nature of immersive sport spectatorship.

Overall, this review contributes to the development of a more cumulative behavioral science of immersive sport spectatorship by clarifying the current state of the literature, identifying major research gaps, and outlining future research directions. Future studies should build integrated models that connect technology design, psychological mechanisms, contextual moderators, and spectator outcomes. Such work can help researchers explain how immersive technologies shape sport spectator behavior intentions and aid practitioners in designing immersive viewing experiences that are innovative, usable, meaningful, and psychologically sustainable.

## Figures and Tables

**Table 1:** The search query for databases.

Database	Search Query
Scopus 142/383	(TITLE-ABS-KEY (“augmented reality” OR AR OR “virtual reality” OR VR OR “360-degree” OR “extended reality” OR XR OR “mixed reality” OR MR OR “immersive media” OR “immersive video” OR “spatial computing”) AND TITLE-ABS-KEY (sport*) AND TITLE-ABS-KEY (spectator* OR view* OR watch* OR fan* OR audience*) AND TITLE-ABS-KEY (experience* OR spectatorship OR behavior* OR intention* OR engagement* OR behaviour*) AND (LIMIT-TO (DOCTYPE, “ar”)) AND (LIMIT-TO (LANGUAGE, “English”)) AND (LIMIT-TO (SRCTYPE, “j”))
Web of Science 116/255	(TS = (“augmented reality” OR AR OR “virtual reality” OR VR OR “360-degree” OR “extended reality” OR XR OR “mixed reality” OR MR OR “immersive media” OR “immersive video” OR “spatial computing”) AND TS = (sport*) AND TS = (spectator* OR view* OR watch* OR fan* OR audience*) AND TS = (experience* OR spectatorship OR behavior* OR intention* OR engagement* OR behaviour*) AND (DT = (“ARTICLE”) AND LA = (“ENGLISH”))
ProQuest 97/204	abstract (“augmented reality” OR AR OR “virtual reality” OR VR OR “360-degree” OR “extended reality” OR XR OR “mixed reality” OR MR OR “immersive media” OR “immersive video” OR “spatial computing) AND abstract (sport*) AND abstract (spectator* OR view* OR watch* OR fan* OR audience*) AND abstract (experience* OR spectatorship OR behavior* OR intention* OR engagement* OR behaviour*) AND (la.exact (“ENG”) AND stype.exact (“Scholarly Journals”) AND PEER (yes))

**Table 2:** Publication journal.

Journal	Articles
*International Journal of Sports Marketing and Sponsorship*	5
*Computers in Human Behavior*	4
*Sport Marketing Quarterly*	3
*Frontiers in Virtual Reality*	2
*Communication & Sport*	1
*Computers & Graphics*	1
*Current Issues in Tourism*	1
*Event Management*	1
*IEEE Transactions on Visualization and Computer Graphics*	1
*International Journal of Consumer Studies*	1
*Journal of Asian Scientific Research*	1
*Journal of Broadcasting & Electronic Media*	1
*Journal of Consumer Behaviour*	1
*Journal of Global Sport Management*	1
*Journal of Innovation & Knowledge*	1
*Journal of Promotion Management*	1
*Journal of Research in Interactive Marketing*	1
*Journal of Sport Management*	1
*Journal of Sports Media*	1
*Journal of Theoretical and Applied Electronic Commerce Research*	1
*Media Watch*	1
*Sport Management Review*	1
*Sport, Business and Management: An International Journal*	1
*Sustainability*	1

**Table 3:** Participant characteristics of the included articles.

Participant Category	Sample Population	Articles
University samples	University students	17
General population samples	General population; general audience with prior sports-watching experience	3
Sport-related participant samples	Baseball fans/rugby fans/soccer fans/sport fans/sport spectators/ sport enthusiasts/sport consumers	8
Experience or context specific samples	Users with prior VR sports-viewing experience; Beijing Winter Olympics attendees; Beijing Winter Olympics attendees who had used smartphone AR while watching the events	3
Mixed or comparative participant samples	University students and match attendees; football fans and non-football fans; university students and sports viewers	3

**Table 4:** Theories and models used in the included articles.

Technology Type	Theory or Model	Articles
VR, AR, and MR	Technology acceptance model (TAM)	7
	Flow theory	6
	Biased competition model	3
	Elaboration likelihood model	2
	Stimulus–organism–response framework (S–O–R)	2
VR & 360	Limited capacity model of motivated mediated message processing	2
VR	Telepresence theory	4
	Diffusion of innovation theory	2
	Dynamic human-centered communication system theory	2
	Unified theory of acceptance and use of technology 2 (UTAUT2)	1
	Uses and gratifications theory	2
	Affective expectation theory	1
	Attachment theory	1
	Construal level theory	1
	Emotion dynamics framework	1
	Expectancy value theory	1
	Grounded cognition theory	1
	Hedonic adaptation theory	1
	Image transfer theory	1
	Integrated technology adoption paradigm	1
	Poison theory	1
	Presence theory	1
	Satiation theory	1
	Technology acceptance model 3 (TAM3)	1
	Theory of planned behavior	1
AR	Dual-process theory of habituation and sensitization	2
	Motivated consumer innovativeness framework	2
	Experience economy theory	2
	Self-determination theory	2
	Experiential marketing theory	1
	Habituation theory	1
	Innovation resistance theory	1
	Pleasure–arousal model	1
	Selective attention theory	1
	Sensory information capacity theory	1
	Technology readiness and acceptance model	1
MR	Social identity theory	1

**Table 5:** Technology-wise breakdown of theme (*n* = 34).

Theme	AR	VR	360-Degree	MR	Total
Adoption or resistance to immersive technology and the application of TAMs	2	8	0	1	11
Immersive technology experience and psychological mechanisms	10	12	1	0	23
Total	12	20	1	1	34

**Table 6:** Research on adoption and resistance.

Author	Types	Antecedent Variables	Response Variables	Outcome Variables
Peng and Oh (2024)	VR	Perceived usefulness; Perceived enjoyment; Perceived ease of use; Economic value;	Subjective norms; Satisfaction	Continuance behavioral intention
Goebert and Greenhalgh (2020)	AR	Technology readiness; Visual appeal; Facilitating conditions;	Perceived ease of use; Perceived usefulness; Attitude toward AR	AR usage intention; Word of mouth
Langaro et al. (2022)	VR	Subjective norms; Involvement; Information consumption; Social interactions; Interest curiosity; Gender; Past use	Perceived usefulness; Perceived ease of use; Perceived enjoyment; Attitude toward use; Attitude toward purchase	Attitude toward price; Purchase intention; Use intention
Capasa et al. (2022)	VR	Subjective norm; Output quality; Experience; Price value; Curiosity; Self-construal	Image; Perceived enjoyment; Perceived ease of use; Perceived usefulness; Usage attitude	Intention to use VR
Kunz and Santomier (2020)	VR	Flow; Content quality; Social influence; Facilitating conditions; Hedonic motivation; Price value; Habit	Performance expectancy; Effort expectancy	Behavioral intention
Rynarzewska (2018)	VR	Information consumption frequency; Expected improvement in information consumption; Attitude toward VR		Likelihood of VR adoption
Kang (2020)	VR	Technology fluidity; Computer ownership; VR tool ownership; Audience factors; Innovativeness; Use factors; Social factors; System factors		Adoption belief; Adoption attitude; VRS system adoption
Park et al. (2025)	VR	Innate innovativeness; Actualized innovativeness; Relative advantage; Complexity; Perceived popularity; Companionship; Solitude		VRS adoption status: adopters, non-adopters; Adopter category: continuers, discontinuers, potentials, resistor
Uhlendorf and Uhrich (2024)	AR	Distraction from the live experience; Interference with fan rituals supporting the team; Reduced social interactions; Reduced emotionality in discussions; Risk of personal image damage; Loss of stadium atmosphere; Fan identity incongruence		Postponement; Rejection; Opposition;
Burkett et al. (2026)	VR	Technology innovation	Perceived usefulness; Perceived ease of use; Perceived enjoyment	Flow experience
Selvaratnam et al. (2026)	MR	Private evaluation; Public evaluation; Sense of interdependence with the group; Interconnection of self with the group; Behavioral involvement; Cognitive awareness	Perceived ease of use; Perceived usefulness; Attitude	Purchase intention

Note: A response variable refers to a variable positioned between the antecedent variable and the outcome variable in the original research model. However, this does not necessarily mean that the original study formally tested it as a mediating variable.

**Table 7:** Antecedent variables examined in immersive sport spectatorship research.

Category	Variable	References
Technology type	Broadcast type (traditional broadcast vs. coach-mode broadcast vs. mascot-mode broadcast); Media type: 2D vs. VR; AR vs. non-AR; actual AR vs. indirect AR; 2D screen vs. VR without interruptions vs. VR with interruptions; Player interaction condition: VR vs. real touch; VR broadcasting condition: baseline condition vs. visualization-only condition vs. full-integration condition; Camera types: high-tech cameras vs. non-high-tech cameras; Google Glass	Ahn and Ko (2024); Frewen and Vincent (2024); Goebert et al. (2022); Jang et al. (2022); D. Kim and Ko (2019); Y. Kim and Ahn (2026); Lee and Zhou (2025); Lo et al. (2022); Oh et al. (2022); Paek et al. (2025); Rogers et al. (2017); Vincent and Frewen (2023); Walton (2025); Yao et al. (2026); Petit et al. (2023)
Technology features	Novelty Vividness Informativeness Interactivity Visual appeal	Du et al. (2024); Jang et al. (2022); D. Kim and Ko (2019); Lei and Ngan (2024)
Sensory imagery	Visual imagery Aural imagery Touch imagery	Petit et al. (2023); Chung et al. (2025)
Motivation	Functionally motivated innovativeness; Hedonically motivated innovativeness; Cognitively motivated innovativeness; Socially motivated innovativeness	S. Kim and Manoli (2024)

**Table 8:** Experience-related variables.

Category	Variable	References
Immersive experience	Presence (spatial, temporal, social)	Chung et al. (2025); Frewen and Vincent (2024); Jang et al. (2022); Lee and Zhou (2025); Lo et al. (2022); Vincent and Frewen (2023)
	Telepresence	D. Kim and Ko (2019); Oh et al. (2022); Walton (2025)
	Immersion	Petit et al. (2023); Yao et al. (2026)
	Flow experience	Chang et al. (2024); D. Kim and Ko (2019); Oh et al. (2022); Kunz and Santomier (2020)
Quality of experience	Comfort	Yao et al. (2026)
	User experience	Lo et al. (2022)
Multidimensional experiences	Aesthetic experience; Educational experience; Entertainment experience; Escapist experience;	Du et al. (2024)
	Engagement (cognitive, affective, physical, novelty)	Y. Kim and Ahn (2026); Lei and Ngan (2024); Ngan and Lei (2025); Yao et al. (2026)

**Table 9:** Psychological response variables.

Category	Variable	References
Cognition	Cognitive engagement	Ngan and Lei (2025); Lei and Ngan (2024)
	Cognitive resource allocation	Lee and Zhou (2025); Paek et al. (2025)
	Visual attention	Paek et al. (2025)
	Brand recall; Brand recognition; Recognition sensitivity	Lee and Zhou (2025); Paek et al. (2025); Walton (2025)
	Cognitive absorption	Ahn and Ko (2024)
	Perceived visual stimuli; Perceived aural stimuli	Chung et al. (2025)
	Perceived usefulness; Perceived ease of use	Lei and Ngan (2024)
	Comprehension Spatial understanding Game understanding	Lo et al. (2022); Yao et al. (2026)
	Workload	Lo et al. (2022)
	Psychological distance	Petit et al. (2023)
Affective	Affective engagement;	Ngan and Lei (2025); Lei and Ngan (2024)
	Enjoyment	Ahn and Ko (2024); Jang et al. (2022); Rogers et al. (2017)
	Arousal	Chang et al. (2024); Chung et al. (2025); Lee and Zhou (2025); Uhm et al. (2020)
	Emotion dynamics	Chang et al. (2024)
	Team attachment	Petit et al. (2023)
Satisfaction	Satisfaction	Chang et al. (2024); Frewen and Vincent (2024); D. Kim and Ko (2019); S. Kim and Manoli (2024); Lo et al. (2022); Oh et al. (2022); Vincent and Frewen (2023)
Attitude	Attitude (hedonic; utilitarian)	Goebert et al. (2022); Lei and Ngan (2024); Uhm et al. (2020)
Psychological needs	Relatedness; Autonomy; Competence	Rogers et al. (2017)

**Table 10:** Negative experience-related variables.

Category	Variable	References
Negative experience	Irritation	Ahn and Ko (2024)
	Distraction	Jang et al. (2022)
	Cognitive load	Rogers et al. (2017)

**Table 11:** Behavioral intention variables.

Category	Variable	References
Media and content engagement intentions	Intention to follow social media	Jang et al. (2022)
	Intention to watch again	Du et al. (2024); Goebert et al. (2022)
	Stickiness	Ahn and Ko (2024)
	Media engagement intention	Y. Kim and Ahn (2026)
Consumption intentions	Consumption intention	Chung et al. (2025)
	Intention to pay	Du et al. (2024)
Advocacy and communication intentions	Intention to recommend	Du et al. (2024)
	Word of mouth (positive; negative)	Goebert et al. (2022); S. Kim and Manoli (2024); Lei and Ngan (2024)
Revisit intentions	Revisit intention	Ngan and Lei (2025)
	Future event visiting intention	Lei and Ngan (2024)

**Table 12:** Moderating variables.

Category	Variable	References
Individual characteristics	Sport involvement	Ahn and Ko (2024); Goebert et al. (2022); Jang et al. (2022); D. Kim and Ko (2019); Y. Kim and Ahn (2026)
	Hedonic innovativeness	Y. Kim and Ahn (2026)
	Past VR experience	Chang et al. (2024)
	Need for cognition	Rogers et al. (2017)
Sport context characteristics	Game situation: (close game vs. decisive game)	Chang et al. (2024)
	Game rivalry	D. Kim and Ko (2019)

**Table 13:** Future research agenda for immersive sport spectatorship.

Possible Research Focus	Future Direction	Research Gap
Distinguish technological immersion from presence, telepresence, flow, engagement, enjoyment, and satisfaction	Clarify and integrate key concepts	Conceptual ambiguity
Connect TAM-based constructs with presence, flow, attention, cognitive load, satisfaction, resistance, and behavioral reasoning theory	Integrate adoption, experience, cognition, affect, and resistance	Fragmented theoretical models
Test different immersive technologies under comparable sport-viewing conditions	Compare VR, AR, MR and 360-degree video	Uneven technology coverage
Examine casual viewers, highly identified fans, older adults, disabled spectators, international fans, and different sport types	Broaden samples, sports, and cultural settings	Limited contextual diversity
Use interviews, diary studies, field experiments, repeated exposure designs, and platform data	Expand qualitative, longitudinal, field-based, and mixed-methods designs	Methodological concentration
Combine self-reports with eye tracking, physiological measures, behavioral logs, interaction data, and actual post-viewing behavior	Use multimodal indicators	Measurement limitations
Study irritation, distraction, cognitive load, discomfort, resistance, discontinuance, habituation, and long-term engagement	Examine boundary conditions and sustainable use	Limited negative-outcome research

## Data Availability

The original contributions presented in the study are included in the main text and the Appendix A. Further inquiries can be directed to the corresponding author.

## Appendix A

**Table A1:** Characteristics of the included articles.

Author(s)/Year	Journal	Technology Type	Device/Interface	Sport and Viewing Context	Studies (*n*)	Country	Research Methods	Data Collection
Burkett et al. (2026)	*Journal of Innovation & Knowledge*	VR	VR headset (360° live stream) and smartphone streaming application; direct device use	Professional basketball (Movistar Estudiantes vs. Super Agropal Palencia); live stream of the live match; on-site venue-based viewing	1	Spain	Quantitative	Post-exposure survey
Y. Kim and Ahn (2026)	*Computers in Human Behavior*	AR	2D screen displaying AR-enhanced and non-AR broadcast videos; no dedicated AR hardware used	NFL football (2021–2022 Wild Card game, San Francisco 49ers vs. Dallas Cowboys); recorded 3.5-min highlight; laboratory viewing	1	United States	Quantitative	Eye-tracking; Galvanic skin response (GSR); Post-exposure survey
Selvaratnam et al. (2026)	*Sport Marketing Quarterly*	MR	Apple Vision Pro MR headset; no actual device use	General sport media consumption; brief description and video presenting the Apple Vision Pro’s potential for sport viewing; conceptual MR sport-viewing scenario	1	United States	Quantitative	Online survey
Chung et al. (2025)	*International Journal of Sports Marketing and Sponsorship*	VR	Samsung Gear VR HMD; direct device use	Collegiate women’s volleyball (NCAA Division I); recorded 3-min highlights; laboratory viewing	1	United States	Quantitative	Post-exposure survey
Lee and Zhou (2025)	*International Journal of Sports Marketing and Sponsorship*	VR	Oculus Quest VR headset vs. 2D screen; direct device use	European soccer match (2019 UEFA Champions League semi-final, Tottenham Hotspur vs. Ajax Amsterdam); recorded 5-min highlight clip; laboratory viewing	1	United States	Quantitative	Electrodermal activity measures; Cardiac activity measures; Brand memory measures (recognition); Post-exposure survey
Ngan and Lei (2025)	*Current Issues in Tourism*	AR	Smartphone-based AR application; direct on-site AR use	Beijing Winter Olympics; on-site attendance viewing	1	China	Quantitative	Online survey
Paek et al. (2025)	*Communication & Sport*	VR	HTC VIVE VR headset vs. 2D screen; direct device use	NBA basketball (2022 Las Vegas Summer League: Houston Rockets vs. Orlando Magic); recorded 2-min highlight clip; laboratory viewing	1	South Korea	Quantitative	Eye-tracking; Heart rate measurement; Skin conductance measurement; Brand memory measures (recall and recognition); Post-exposure survey
Park et al. (2025)	*International Journal of Sports Marketing and Sponsorship*	VR	Not reported	Virtual reality spectator sport; picture-and brief-explanation-based conceptual VR sport-viewing scenario	1	United States	Quantitative	Post-exposure survey
Walton (2025)	*Sport Marketing Quarterly*	VR	Meta Quest 2 VR HMD vs. 2D screen-based broadcast display; direct device use	Tennis (2021 Australian Open); recorded 5-min clip; laboratory viewing	1	United States	Quantitative	Post-exposure survey
Yao et al. (2026)	*IEEE Transactions on Visualization and Computer Graphics*	VR	Oculus Quest 3 VR HMD; direct VR device use	Tennis (2019 Wimbledon Men’s Final); simulated VR broadcast; laboratory viewing	1	Not reported	Mixed methods	Semi-structured interviews; Post-exposure survey
Ahn and Ko (2024)	*Journal of Sport Management*	AR	2D screen displaying AR-enhanced broadcast video; no dedicated AR hardware used	NFL football (2021–2022 Wild Card game, San Francisco 49ers vs. Dallas Cowboys); recorded highlights; online viewing	1	United States	Quantitative	Online survey
Chang et al. (2024)	*Sport Marketing Quarterly*	VR	Oculus Go VR HMD; direct device use	NBA basketball; recorded 30-min game summaries; laboratory viewing	2	United States	Quantitative	Post-exposure survey
Du et al. (2024)	*Journal of Consumer Behaviour*	AR	2D screen displaying AR-infused sport videos; no dedicated AR hardware used	Professional football (2022 La Liga match, FC Barcelona vs. Cádiz); recorded 3.5-min clips; online and offline viewing	2	China	Mixed methods	Semi-structured interviews; Online survey
Frewen and Vincent (2024)	*Frontiers in Virtual Reality*	VR	Smartphone-mediated VR HMD vs. 2D tablet computer; direct device use	Collegiate volleyball and basketball; live streams vs. recorded 360° streams; on-site field viewing	1	Canada	Quantitative	Post-exposure survey
S. Kim and Manoli (2024)	*International Journal of Sports Marketing and Sponsorship*	AR	Not reported	Professional baseball; AR live streaming; prior actual AR live-streaming viewing	1	South Korea	Quantitative	Online survey
Lei and Ngan (2024)	*Event Management*	AR	Smartphone AR application; direct on-site AR use	Beijing Winter Olympics; AR-assisted live on-site sports-event viewing	1	China	Quantitative	Online survey
Peng and Oh (2024)	*Journal of Asian Scientific Research*	VR	Not reported	VR sports viewing during COVID-19	1	China	Quantitative	Online survey
Uhlendorf and Uhrich (2024)	*Journal of Global Sport Management*	AR	Not reported	Team sports (soccer focus); hypothetical in-stadium AR use	3	Multiple countries	Mixed methods	Semi-structured interviews; Online survey
Vincent and Frewen (2023)	*Frontiers in Virtual Reality*	VR	Smartphone-mediated VR HMD vs. 2D tablet computer; direct device use	Collegiate volleyball and basketball; live streams of university games; field setting	1	Canada	Quantitative	Post-exposure survey
Petit et al. (2023)	*International Journal of Consumer Studies*	VR	VR headset; direct VR device use	Rugby (French Top 14); prerecorded 360° video of the player’s pre-match stadium entry, in-match game phases, and post-match spectator interaction and return to the locker room; dedicated viewing room inside Pau Stadium	2	France	Quantitative	Post-exposure survey
Capasa et al. (2022)	*Journal of Theoretical and Applied Electronic Commerce Research*	VR	Not reported	Mega sports events (Olympic context); explanatory video illustrating prospective VR use; online viewing	1	Multiple countries	Quantitative	Online survey
Goebert et al. (2022)	*Computers in Human Behavior*	AR	2D screen displaying AR-enhanced broadcast video; no dedicated AR hardware used	NBA basketball broadcasts (Los Angeles Clippers vs. Denver Nuggets); recorded 6-min clips (traditional vs. AR-enhanced coach-mode vs. AR-enhanced mascot-mode broadcasts); online viewing	1	United States	Quantitative	Post-exposure survey
Jang et al. (2022)	*International Journal of Sports Marketing and Sponsorship*	360-degree video/camera	2D screen displaying 360-degree video; no direct camera use	NFL football; recorded highlight (high-tech vs. non-high-tech camera footage); online viewing	1	United States	Quantitative	Online survey
Langaro et al. (2022)	*Journal of Promotion Management*	VR	Not reported	Soccer; conceptual VR match-day experience presented through description and images	1	Portugal	Quantitative	Online survey
Lo et al. (2022)	*Computers & Graphics*	AR	Mobile AR interfaces (direct and indirect prototypes); direct AR device use	Rugby; laboratory-based simulated viewing and on-site stadium viewing	2	New Zealand	Quantitative	Post-exposure survey; Task-performance measures
Oh et al. (2022)	*Sustainability*	VR	Oculus Quest 2 VR HMD vs. 2D screen; direct device use	Soccer match (K League All-Star team vs. Juventus FC); recorded 12-min clip; laboratory viewing	1	South Korea	Quantitative	Post-exposure survey
Goebert and Greenhalgh (2020)	*Computers in Human Behavior*	AR	2D screen displaying smartphone-based AR activation prototype videos; no dedicated AR hardware used	College basketball; video demonstrations of smartphone-based AR activations (wayfinding, player interview, and in-game player tracking) in a college basketball arena; online viewing	1	United States	Quantitative	Online survey
Kang (2020)	*Journal of Broadcasting & Electronic Media*	VR	Not reported	General sport spectatorship; no specific sport content or viewing setting	1	United States	Quantitative	Online survey
Kunz and Santomier (2020)	*Sport, Business and Management: An International Journal*	VR	Samsung Gear VR HMD; direct device use	Bundesliga soccer and freestyle skiing; recorded 5-min VR clips; on-site VR viewing	1	Germany	Quantitative	Post-exposure survey
Uhm et al. (2020)	*Sport Management Review*	VR	Samsung New Gear VR headset; direct device use	Luge; recorded first-person perspective 90-s clip; laboratory viewing	1	South Korea	Quantitative	EEG-based neurophysiological measurement; Post-exposure survey
D. Kim and Ko (2019)	*Computers in Human Behavior*	VR	PlayStation VR headset vs. 2D screen; direct device use	NBA basketball; recorded 5-min clip; laboratory viewing	1	United States	Quantitative	Post-exposure survey
Rogers et al. (2019)	*Media Watch*	AR	Google Glass; direct device use	College basketball; recorded 10-min clip; theater-based group viewing	1	United States	Qualitative	Post-exposure open-ended questionnaire
Rynarzewska (2018)	*Journal of Research in Interactive Marketing*	VR	Conventional screen displaying video; no direct VR use	General sport consumption; recorded 75-s clips; promotional video presenting VR headset-based 360-degree customizable viewing of games, selected footage, practices, and exclusive team content; online viewing	1	United States	Quantitative	Online survey
Rogers et al. (2017)	*Journal of Sports Media*	AR	Google Glass; direct device use	College basketball; recorded 10-min clip; theater-based group viewing	1	United States	Quantitative	Post-exposure survey

**Table A2:** Participant characteristics and sample information of the included articles.

Author(s)/Year	Participant Characteristics	Sample (*n*)	Age	Gender	Prior VR/AR/MR/360 Experience Reported
Burkett et al. (2026)	University students and match attendees	81	Reported	Reported	Reported
Y. Kim and Ahn (2026)	University students	156	Reported	Reported	Reported
Selvaratnam et al. (2026)	Sport fans	272	Reported	Reported	Not reported
Chung et al. (2025)	University students	131	Reported	Reported	Reported
Lee and Zhou (2025)	University students	40	Reported	Reported	Not reported
Ngan and Lei (2025)	Beijing Winter Olympics attendees	452	Reported	Reported	Reported
Paek et al. (2025)	University students	57	Reported	Reported	Not reported
Park et al. (2025)	University students	216	Reported	Reported	Reported
Walton (2025)	University students	66	Not reported	Not reported	Not reported
Yao et al. (2026)	University students	16	Reported	Reported	Reported
Ahn and Ko (2024)	Football fans = 244; Non-football fans = 172	416	Reported	Reported	Reported
Chang et al. (2024)	University students	234 (Study 1 = 74; Study 2 = 160)	Reported	Reported	Reported
Du et al. (2024)	Study 1: University students Study 2: Sports viewers	342 (Study 1 = 40; Study 2 = 302)	Reported	Reported	Study 1: Reported Study 2: Not reported
Frewen and Vincent (2024)	University students	148	Not reported	Reported	Reported
S. Kim and Manoli (2024)	Baseball fans	243	Reported	Reported	Reported
Lei and Ngan (2024)	Beijing Winter Olympics attendees who had used smartphone AR while watching the events	204	Reported	Reported	Reported
Peng and Oh (2024)	Users with prior VR sports-viewing experience	262	Reported	Reported	Reported
Uhlendorf and Uhrich (2024)	Sport spectators	1794 (Study 1 = 22; Study 2 = 566; Study 3 = 1206)	Reported	Reported	Reported
Vincent and Frewen (2023)	University students	93	Not reported	Reported	Not reported
Petit et al. (2023)	Section Paloise rugby supporters	241 (Study 1 = 146; Study 2 = 95)	Reported	Reported	Not reported
Capasa et al. (2022)	Sport enthusiasts	339	Reported	Reported	Not reported
Goebert et al. (2022)	General audience with prior sports watching experience	212	Reported	Reported	Not reported
Jang et al. (2022)	Sport consumers	289	Reported	Reported	Not reported
Langaro et al. (2022)	Soccer fans	203	Reported	Reported	Reported
Lo et al. (2022)	University students	46 (Study 1 = 30; Study 2 = 16)	Reported	Reported	Reported
Oh et al. (2022)	University students	149	Reported	Reported	Not reported
Goebert and Greenhalgh (2020)	Sport fans	142	Reported	Reported	Not reported
Kang (2020)	General population	488	Reported	Reported	Not reported
Kunz and Santomier (2020)	University students	518	Reported	Reported	Reported
Uhm et al. (2020)	General population	36	Reported	Reported	Not reported
D. Kim and Ko (2019)	University students	129	Reported	Reported	Not reported
Rogers et al. (2019)	University students	65	Reported	Reported	Not reported
Rynarzewska (2018)	University students	77	Reported	Reported	Not reported
Rogers et al. (2017)	University students	65	Reported	Reported	Reported

**Table A3:** Theoretical frameworks, variable structures, and key findings of the included articles.

Author(s)/Year	Theme	Theoretical/Model	Antecedents	Psychological Response	Outcomes	Moderators	Control Variables	Key Findings
Burkett et al. (2026)	Adoption or resistance to immersive technology and the application of TAMs	Technology Acceptance Model (TAM)	Technology innovation	Perceived usefulness; Perceived ease of use; Perceived enjoyment	Flow experience	Not reported	Not reported	Perceived ease of use, usefulness, and enjoyment enhanced flow in both VR and mobile 360° live streaming (+); no significant differences between the VR and mobile groups (n.s.).
Y. Kim and Ahn (2026)	Immersive experience and psychological responses	Pleasure–Arousal Model; Motivated Consumer Innovativeness (MCI) Framework; Dual-Process Theory of Habituation and Sensitization; Selective Attention Theory; Biased Competition Model; Stimulus–Organism–Response (S–O–R) Framework	Broadcasting type: AR vs. Non-AR	Affective engagement; Cognitive engagement	Media engagement intention	Hedonic innovativeness; Sport involvement	Team identification	AR increased cognitive engagement by directing attention to overlays (+) but had no direct effect on affective engagement or behavioral intention (n.s.); sport involvement strengthened AR effects on affective engagement (+), hedonic innovativeness predicted behavioral intention (+), and repeated exposure produced habituation (affective and cognitive engagement declined).
Selvaratnam et al. (2026)	Adoption or resistance to immersive technology and the application of TAMs	Technology Acceptance Model (TAM); Social Identity Theory (SIT)	Private evaluation; Public evaluation; Sense of interdependence with the group; Interconnection of self with the group; Behavioral involvement; Cognitive awareness	Perceived ease of use; Perceived usefulness; Attitude	Purchase intention	Not reported	Not reported	The extended TAM received partial support; private evaluation increased perceived usefulness (+), public evaluation and interdependence increased perceived ease of use (+), and cognitive awareness decreased perceived usefulness (−); usefulness and ease of use improved attitude, which increased purchase intention (+).
Chung et al. (2025)	Immersive experience and psychological responses	Stimulus–Organism–Response (S–O–R) Framework	Visual imagery; Aural imagery	Perceived visual stimuli; Perceived aural stimuli; Presence; Arousal	Consumption intention	Not reported	Team identification	Sensory imagery and perceived sensory stimuli enhanced presence and arousal, which in turn increased VR consumption intention (all +; S–O–R supported).
Lee and Zhou (2025)	Immersive experience and psychological responses	Dynamic Human-Centered Communication Systems Theory	Media type: VR vs. 2D	Not reported	Arousal; Presence; Cognitive resource allocation; Memory for in-stadium sponsor signage (Recognition sensitivity)	Not reported	Soccer involvement	VR viewing produced higher presence, psychophysiological arousal, and attention than 2D viewing (+) but lower recognition of in-stadium sponsor signage (−).
Ngan and Lei (2025)	Immersive experience and psychological responses	Experiential Marketing Theory	Affective engagement; Cognitive engagement; Novelty engagement; Physical engagement	Not reported	Revisit intention	Not reported	Not reported	Cognitive engagement was higher among AR users and more strongly predicted revisit intention (+); event-experience dimensions increased revisit intention except novelty (n.s.).
Paek et al. (2025)	Immersive experience and psychological responses	Dynamic Human-Centered Communication Systems Theory	Media type: VR vs. 2D; Signage location: central vs. peripheral; Signage-advertising phase: phase1 vs. phase2	Not reported	Cognitive resource allocation (heart rate); Visual attention (Fixation frequency; Fixation duration); Brand recall; Brand recognition; Recognition sensitivity (d′)	Not reported	NBA viewing involvement	VR tended to elicit greater cognitive resource allocation but significantly decreased visual attention to and brand memory for signage advertising (−); centrally placed signage outperformed peripheral placement regardless of media type.
Park et al. (2025)	Adoption or resistance to immersive technology and the application of TAMs	Diffusion of Innovations Theory; Uses and Gratifications Theory	Innovativeness: (1) Innate innovativeness (2) Actualized innovativeness; Perceived characteristics: (1) Relative advantage (2) Complexity; Perceived popularity; Perceived needs: (1) Companionship (2) Solitude; Gender	Not reported	VRS adoption status: Adopters; Non-adopters; Adopter category: Continuers; Discontinuers; Potentials; Resistors	Not reported	Not reported	Actualized innovativeness and the study’s measure labeled complexity (worded as ease of learning and use) predicted adoption; companionship and gender differentiated adopters from non-adopters; adoption factors also differed across continuer, discontinuer, potential, and resistor categories.
Walton (2025)	Immersive experience and psychological responses	Limited Capacity Model of Motivated Mediated Message Processing (LC4MP); Telepresence Theory	Media type: VR vs. 2D	Not reported	Telepresence; Brand recall; Brand recognition	Not reported	Fan identification	VR increased telepresence (+) but reduced brand recall relative to 2D viewing (−), consistent with the LC4MP.
Yao et al. (2026)	Immersive experience and psychological responses	Not reported	VR broadcasting condition: Baseline condition vs. Visualization Only condition vs. Full Integration condition	Not reported	Comprehension; Engagement; Immersion; Comfort	Not reported	Not reported	Full integration of embedded visualizations and adaptive camera techniques improved comprehension, narrative engagement, and immersion relative to baseline VR broadcasting (+) while maintaining viewing comfort.
Ahn and Ko (2024)	Immersive experience and psychological responses	Dual-Process Theory of Habituation and Sensitization; Biased Competition Model; Sensory Information Capacity Theory	Broadcasting type: AR vs. Non-AR; Highlight Length: brief vs. prolonged	Not reported	Cognitive absorption; Enjoyment; Irritation; Stickiness	Sport involvement	Past game-watching experience; San Francisco 49ers team identification; Dallas Cowboys team identification; Prior AR experience; Technology readiness	Highly involved viewers showed habituation to prolonged AR highlights, whereas less involved viewers showed sensitization to both brief and prolonged AR highlights; enjoyment and irritation varied by involvement and exposure duration (conditional effects).
Chang et al. (2024)	Immersive experience and psychological responses	Flow Theory; Affective Expectation Theory; Biased Competition Model; Emotion Dynamics Framework; Poison theory	Study 1: Spectatorship involvement (low vs. high); Study 2: Flow experiences (little vs. abundant)	Study 2: Emotion dynamics (arousal and valence)	Study 1: Flow experiences; Study 2: Spectatorship satisfaction	Study 1: Past VR experience; Study 2: Game situation (close game vs. decisive game)	Study 1: Team identification; emotion reactivity; Study 2: Not reported	Lower spectatorship involvement and less prior VR experience increased VR flow (Study 1); VR flow enhanced satisfaction in both close and decisive games via emotion dynamics (Study 2) (+).
Du et al. (2024)	Immersive experience and psychological responses	Experience Economy Theory	Novelty; Vividness; Informativeness	Entertainment experience; Educational experience; Aesthetic experience; Escapist experience	Intention to watch again; Intention to recommend; Intention to pay	Not reported	Not reported	AR novelty, vividness, and informativeness shaped entertainment, educational, aesthetic, and escapist experiences, which increased intentions to rewatch, recommend, and pay (+).
Frewen and Vincent (2024)	Immersive experience and psychological responses	Not reported	Device Type (3D-VR vs. 2D); Viewing time (live vs. playback)	Not reported	Spatial presence; Temporal presence; Social presence; Satisfaction	Not reported	Not reported	3D-VR viewing increased spatial, interpersonal, and temporal presence relative to 2D-tablet viewing (+); live viewing increased temporal presence relative to recorded viewing (+).
S. Kim and Manoli (2024)	Immersive experience and psychological responses	Motivated Consumer Innovativeness (MCI) Framework	Functionally Motivated Consumer Innovativeness; Hedonically Motivated Consumer Innovativeness; Cognitively Motivated Consumer Innovativeness; Socially Motivated Consumer Innovativeness	Satisfaction	Positive word-of-mouth	Not reported	Game satisfaction	Functional, hedonic, and cognitive innovativeness increased positive word-of-mouth directly and via satisfaction (+); socially motivated innovativeness influenced word-of-mouth only through satisfaction (full mediation).
Lei and Ngan (2024)	Immersive experience and psychological responses	Technology Acceptance Model (TAM); Experience Economy Theory	Visual appeal; Perceived usefulness; Perceived ease of use;	Attitude toward AR; Affective engagement; Cognitive engagement; Physical engagement	Future revisit intention; Positive word-of-mouth; Negative word-of-mouth	Not reported	Not reported	AR usefulness and ease of use enhanced physical, cognitive, and affective engagement, which increased revisit intention and positive word-of-mouth (+).
Peng and Oh (2024)	Adoption or resistance to immersive technology and the application of TAMs	Technology Acceptance Model (TAM); Theory of Planned Behavior (TPB)	Perceived usefulness; Perceived enjoyment; Perceived ease of use; Economical value	Subjective norms; Consumer satisfaction	Continuance behavioral intention	Not reported	Not reported	Perceived value dimensions (usefulness, enjoyment, ease of use, economic value) increased subjective norms and consumer satisfaction, both of which increased the intention to continue using VR for sports viewing (+).
Uhlendorf and Uhrich (2024)	Adoption or resistance to immersive technology and the application of TAMs	Innovation Resistance Theory	Distraction from the live experience; Interference with fan rituals supporting the team; Reduced social interactions; Reduced emotionality in discussions; Risk of personal image damage; Loss of stadium atmosphere; Fan identity incongruence	Not reported	Postponement; Rejection; Opposition	Not reported	Not reported	Existing innovation resistance barriers were contextualized into seven sport-specific AR adoption barriers, which subsequently increased spectators’ postponement (+), rejection (+), and opposition (+) toward in-stadium AR.
Vincent and Frewen (2023)	Immersive experience and psychological responses	Not reported	Device type (VR vs. 2D)	Not reported	Spatial presence; Temporal presence; Social presence; Satisfaction	Not reported	Not reported	3D-VR viewing increased spatial, interpersonal, and temporal presence compared with standard viewing (+), and participants generally preferred VR viewing.
Petit et al. (2023)	Immersive experience and psychological responses	Grounded Cognition Theory; Telepresence Theory; Attachment Theory; Image Transfer Theory	Study 1: Touch imagery; Study 2: Player interaction condition (VR vs. real touch)	Study 1: Psychological distance from the player; Team attachment. Study 2: Immersion; Touch imagery; Psychological distance from the player; Team attachment	Sponsor attachment	Not reported	Not reported	Study 1: Touch imagery enhanced sponsor attachment through psychological distance and team attachment. Study 2: VR, compared with real touch, enhanced sponsor attachment through immersion, touch imagery, psychological distance, and team attachment.
Capasa et al. (2022)	Adoption or resistance to immersive technology and the application of TAMs	Technology Acceptance Model 3 (TAM3); Construal Level Theory (CLT)	Subjective norm; Output quality; Experience; Price value; Curiosity; Self-construal	Image; Perceived enjoyment; Perceived ease of use; Perceived usefulness; Usage attitude	Intention to use VR	Not reported	Not reported	Performance expectancy, effort expectancy, social influence, curiosity, and self-construal all increased the intention to use VR for spectating mega sports events (+).
Goebert et al. (2022)	Immersive experience and psychological responses	Elaboration Likelihood Model (ELM)	Broadcast type: traditional vs. coach mode vs. mascot mode	Not reported	Hedonic attitude; Utilitarian attitude; Re-viewing intention; Word-of-mouth	Sport involvement	Sport involvement	Word-of-mouth intention was lower for the mascot-mode AR broadcast than for traditional and coach-mode broadcasts (−); utilitarian and hedonic attitudes varied partially across broadcast types; sport involvement did not significantly moderate these effects (n.s.).
Jang et al. (2022)	Immersive experience and psychological responses	Limited Capacity Model of Motivated Mediated Message Processing (LC4MP)	Camera types: High-tech cameras vs. Non-high-tech cameras	Perceived novelty; Perceived distraction	Presence; Enjoyment; Intention to follow the YouTube channel	Sport involvement	Team identification	High-tech camera footage increased perceived novelty and social-media follow intention for highly involved consumers (+) but distracted less involved consumers, whose intentions were not strengthened (conditional).
Langaro et al. (2022)	Adoption or resistance to immersive technology and the application of TAMs	Technology Acceptance Model (TAM)	Subjective Norms; Involvement; Information Consumption; Social Interactions; Interest Curiosity; Gender; Past Use	Perceived usefulness; Perceived ease of use; Perceived enjoyment; Attitude toward purchase; Attitude toward use	Attitude toward price; Purchase intention; Use Intention	Not reported	Not reported	Perceived usefulness, perceived enjoyment, and attitudes strongly predicted intentions to purchase and use VR (+).
Lo et al. (2022)	Immersive experience and psychological responses	Not reported	Study 1: Visualization method: traditional infographics (TI) vs. situated infographics (SI). Study 2a: Actual AR vs. Indirect AR; Study 2b: Visualization methods: situated broadcast-style visualizations (SBV) vs. situated infographics (SI) Study 2c: Unassisted on-site viewing vs. SBV vs. SI vs. television broadcasting	Not reported	Workload; Spatial understanding; Game understanding; Presence; User experience; User preference; Team fanaticism; Task performance; Satisfaction	Not reported	Not reported	Situated visualizations improved game understanding and enriched the on-site spectator experience (+) without increasing overall workload (n.s.); the visualization technique influenced spatial understanding.
Oh et al. (2022)	Immersive experience and psychological responses	Telepresence Theory; Flow Theory; Hedonic Adaptation Theory; Satiation Theory	Viewing type: 2D screen vs. VR without interruptions vs. VR with interruptions (4, 8, and 12 min)	Not reported	Telepresence; Flow experience; Spectatorship satisfaction	Not reported	Sport involvement; team identification	VRS produced higher telepresence, flow, and satisfaction than a 2D screen (+); interruptions enhanced flow, and satisfaction increased over time in VRS with interruptions (satiation reduced).
Goebert and Greenhalgh (2020)	Adoption or resistance to immersive technology and the application of TAMs	Technology Readiness and Acceptance Model (TRAM); Technology Acceptance Model (TAM)	Technology readiness; Visual appeal; Facilitating conditions	Perceived usefulness; Perceived ease of use; AR attitude	AR usage intention; Word-of-mouth	Not reported	Not reported	Visual appeal increased intention to use AR activations and positive word-of-mouth (+); perceived ease of use was not significant (n.s.).
Kang (2020)	Adoption or resistance to immersive technology and the application of TAMs	Integrated Technology Adoption Paradigm (ITAP); Uses and Gratifications Theory; Diffusion of Innovations Theory	Technology fluidity; Computer ownership; VR tool ownership; Audience factors; Innovativeness; Use factors; Social factors; System factors	Not reported	Adoption Belief; Adoption Attitude; VRS System Adoption	Not reported	Gender; Age; Income; Education; General media use (Television, Radio, Print, Internet, Mobile Phone); Sports-media use (TV Sports, Radio Sports, Print Sports, Internet Sports, Mobile Sports)	Personal factors predicted adoption belief, attitudinal factors predicted adoption attitude, and VR tool ownership and social-network influence directly predicted VRS adoption (+).
Kunz and Santomier (2020)	Adoption or resistance to immersive technology and the application of TAMs	Unified Theory of Acceptance and Use of Technology 2 (UTAUT2); Flow Theory	Flow; Content quality; Social influence; Facilitating conditions; Hedonic motivation; Price value; Habit	Performance expectancy; Effort expectancy	Behavioral intention to use sport VR	Not reported	Not reported	Flow and content quality increased performance and effort expectancy, which, together with UTAUT2 factors, increased behavioral intention to use sport VR (+).
Uhm et al. (2020)	Immersive experience and psychological responses	Presence Theory; Flow Theory	Cognitive involvement stimulus (informational poster exposure vs. no exposure)	Not reported	Arousal; Attitude toward luge	Not reported	Not reported	The presence-stimulated VR group showed higher neurophysiological arousal and a more positive attitude toward the sport (+).
D. Kim and Ko (2019)	Immersive experience and psychological responses	Telepresence Theory; Flow Theory; Elaboration Likelihood Model	Media type: VR vs. 2D;Vividness; Interactivity	Telepresence; Flow experience	Satisfaction	Sport involvement; Game rivalry	Not reported	VR enhanced vividness (+) and interactivity (+), which increased telepresence (+) and subsequently improved flow experience (+) and satisfaction (+). Telepresence functioned as a key psychological mechanism linking VR affordances to flow. Sport involvement positively predicted flow (+) but weakened the indirect effect of VR on flow, indicating stronger VR effects among less involved viewers. Rivalry did not significantly influence flow (n.s.).
Rogers et al. (2019)	Immersive experience and psychological responses	Self-Determination Theory (SDT); Flow Theory	Not applicable	Not applicable	Not applicable	Not applicable	Not applicable	Google Glass added novelty but also distraction and discomfort; enjoyment depended on accessing information with minimal disruption to viewing (qualitative).
Rynarzewska (2018)	Adoption or resistance to immersive technology and the application of TAMs	Technology Acceptance Model (TAM); Expectancy Value Theory (EVT)	Information consumption frequency; Expected improvement in information consumption; Attitude toward VR	Not reported	Likelihood of VR adoption	Not reported	Not reported	Attitude toward VR, frequency of sport media consumption, and expected improvement in sport information consumption increased the likelihood of VR adoption (+).
Rogers et al. (2017)	Immersive experience and psychological responses	Self-Determination Theory (SDT)	Information-seeking device: Google Glass AR vs. smartphone vs. printed media guide	Competence; Autonomy; Relatedness; Cognitive load	Enjoyment	Need for cognition	Gender; Age; Race; Prior interest in or knowledge of basketball; Feelings of novelty; Preference for new technology	Technology type had no direct effect on enjoyment (n.s.) but influenced enjoyment indirectly through feelings of competence and autonomy (+).

Note. Not reported = the element was not reported in the original article; Not applicable = the element was not applicable to the study design or analytical approach. In Goebert et al. (2022), sport involvement was examined as a moderator in interaction analyses and included as a covariate in subsequent ANCOVA/MANCOVA models.
